# TBX3 shapes an immunosuppressive microenvironment and induces immunotherapy resistance

**DOI:** 10.7150/thno.103175

**Published:** 2025-01-06

**Authors:** Zhi Liu, Chunyu Zhang, Jiatong Xiao, Yunbo He, Haisu Liang, Jinliang Huang, Zhiyong Cai, Zhenglin Yi, Mingfeng Chen, Yixiao Li, Jun Zhang, Fenglian liu, Peng Ren, Huihuang Li, Jinbo Chen, Benyi Fan, Jiao Hu, Xiongbing Zu, Dingshan Deng

**Affiliations:** 1 Department of Urology, Xiangya Hospital, Central South University, Changsha, China.; 2 Department of Urology, The Second Affiliated Hospital of Guizhou Medical University, Kaili, China.; 3 Department of Urology, Tongji Hospital, Tongji Medical College, Huazhong University of Science and Technology, Wuhan, China.; 4 National Clinical Research Center for Geriatric Disorders and FuRong Laboratory, Xiangya Hospital, Central South University, Changsha, China.; 5 Department of Urology, The second people's Hospital of Hunan province, Changsha, China.; 6 Department of Imaging, The first people's Hospital of Kaili city, Kaili, China.; 7 Department of Urology, The First Affiliated Hospital of Hunan Normal University, Hunan Normal University, Changsha, China.

**Keywords:** TBX3, immunosuppressive microenvironment, CD8+ T cells, fibroblasts, bladder cancer, immunotherapy

## Abstract

**Background:** Identifying biomarkers that predict immunotherapy efficacy and discovering new targets for combination therapies are critical elements for improving the prognosis of bladder cancer (BLCA) patients.

**Methods:** Firstly, we explored the expression patterns of TBX3 in normal and pan-cancer tissues and the correlation between TBX3 and the immune microenvironment using data from multiple public databases. Then, we combined various techniques, including bulk RNA sequencing, single-cell RNA sequencing, high-throughput cytokine arrays, functional experiments, ProcartaPlex multiplex immunoassays and TissueFAXS panoramic tissue quantification assays, to demonstrate that TBX3 shapes an immunosuppressive tumor microenvironment (TME) in BLCA.

**Results:** We identified TBX3 as a key factor associated with the immunosuppressive microenvironment in BLCA through a systematic multi-omics analysis. We found that TBX3 is primarily expressed in malignant cells, where TBX3^high^ tumor cells increase the secretion of TGFβ1, which promotes the infiltration of cancer-associated fibroblasts (CAFs), thereby forming an immunosuppressive microenvironment. We further demonstrated that TBX3 enhances TGFβ1 expression by binding to the TGFβ1 promoter, and blocking TGFβ1 counteracts the immunosuppressive effects of TBX3. Moreover, TBX3 reduced the cancer-killing efficiency of CD8^+^ T cells by decreasing the proportion of GZMB^+^ CD8^+^ T cells, and knocking down TBX3 combined with anti-PD-1 treatment increased CD8^+^ T cell infiltration and reduced CAFs *in vivo*. We also validated the inverse relationship between TBX3^+^ malignant cells and CD8^+^ T cells and the positive relationship with CAFs in tissue microarrays. Lastly, we found that TBX3 predicted immunotherapy efficacy in our real-world immunotherapy cohort and multiple public cohorts.

**Conclusion:** In summary, TBX3 promotes BLCA progression and immunotherapy resistance by inducing an immunosuppressive microenvironment, and targeting TBX3 could enhance the efficacy of immunotherapy for BLCA.

## Introduction

Bladder cancer (BLCA) is one of the most prevalent carcinomas worldwide, with 613,791 new cases and 220,349 deaths reported in 2022 1. Only approximately 25% of BLCA patients are diagnosed with muscle-invasive bladder cancer (MIBC). Although the majority of patients are initially diagnosed with non-muscle invasive bladder cancer (NMIBC), 15% to 20% of those with NMIBC progress to MIBC, a condition generally considered incurable 2,3. Due to its high tumor mutation burden (TMB), BLCA patients exhibit partial sensitivity to immune checkpoint blockade (ICB)-based immunotherapy, leading to significant advancements in recent years 4,5. However, not all BLCA patients respond to immunotherapy; in fact, up to 80% of patients do not respond, presenting a significant challenge in the treatment of BLCA 6-10. Numerous studies have focused on identifying biomarkers that predict immunotherapy response or discover new targets to overcome immunotherapy resistance. Unfortunately, all these efforts have encountered significant challenges when translated into clinical applications, including PD-L1 testing 11.

Studies over the past decade have shown that stromal cells and the extracellular matrix (ECM) within the tumor microenvironment (TME) co-evolve with cancer cells through mutual interactions, promoting both cancer progression and drug resistance 12,13. The dynamic communication between tumor cells and the TME is considered a crucial driving factor at almost every stage of tumor progression, from local invasion of the primary tumor to distant metastatic colonization 14. Cancer-associated fibroblasts (CAFs), often referred to as activated or reactive fibroblasts surrounding cancer cells 15, are the primary cellular component of the TME. CAFs are deposited in the tumor stroma, serving both as a physical barrier preventing immune cell infiltration and as a structural scaffold for cell-cell interactions, thereby regulating tumorigenesis, angiogenesis, and immune evasion 16-18. However, matrix metalloproteinases produced by CAFs degrade the ECM, leading to interactions between vascular endothelial growth factor A and its receptor, promoting angiogenesis 19. In addition, CAFs secrete substantial amounts of growth factors, proinflammatory cytokines, and chemokines, particularly TGF-β, IL-6, and CC-chemokine ligand 2, which recruit immunosuppressive cells into the tumor stroma and lead to immune escape 20-22. However, how cancer cells regulate CAFs to form an immunosuppressive microenvironment in BLCA is not well understood.

T-box genes are primarily known for their essential roles in embryonic development and organ formation processes 23. In recent years, an increasing number of studies have found that the T-box family of proteins promote cancer initiation and invasiveness either through epithelial-mesenchymal transition (EMT) or by enhancing stemness in cancer cells 24. As a member of the T-box family, TBX3 plays significant roles in cancer development 23. TBX3 has been reported to be abnormally expressed in various carcinomas, including breast, bladder, liver, and pancreatic carcinomas 25-28. Further, TBX3 has been shown to lead to uncontrolled cell proliferation and evasion of senescence and apoptosis, thereby promoting cancer initiation, angiogenesis, and metastasis 29. In BLCA, the methylation of TBX2 and TBX3 has been reported to predict the progression of pTa BLCA 30. Du *et al.* found that TBX3 functions as a critical regulator of E-cadherin expression during BLCA progression 31, and Huang *et al.* revealed that TBX3 promotes BLCA progression by inhibiting apoptosis and increasing cell stemness 32. However, the role of TBX3 in regulating the TME immune status is less studied. In this study, we focused on TBX3 in the TME of BLCA via a pan-cancer analysis and then further investigated its key role in BLCA using multi-omics analysis. We found that TBX3^high^ tumor cells increased the secretion of TGFβ1, thereby promoting infiltration of CAFs and inducing an immunosuppressive microenvironment in BLCA. Further, knocking down TBX3 synergized with anti-PD-1 treatment *in vivo*. Meanwhile, combining our own data with multiple public real-world immunotherapy cohorts, we found that TBX3 expression was associated with immunotherapy resistance.

## Methods

### Collection of multiple datasets

We collected 57 qualified bladder tissue samples along with 13 paired normal samples and subsequently performed bulk RNA sequencing (RNA-seq). This cohort has been uploaded to the GEO database (accession number: GSE188715) and is referred to as the Xiangya cohort 33,34. Pan-cancer analysis data from The Cancer Genome Atlas (TCGA) were downloaded from UCSC Xena (https://xenabrowser.net/), and the FPKM data were converted into TPM data specific to TCGA-BLCA. The BLCA gene expression array data, including GSE13507 and GSE48075, were obtained from the GEO database using the “GEOquery” R function. For immunotherapy cohorts, the IMvigor210 cohort was downloaded from the study by Mariathasan *et al.* 35 (http://research-pub.gene.com/IMvigor210CoreBiologies/ packageVersions/). Additional immunotherapy cohorts, including GSE135222 and GSE173839, were downloaded using the “GEOquery” R function, and the dataset PMID26359337 was obtained from the study by Van *et al.* 36.

### Tissue specimen and immunohistochemistry (IHC)

We obtained 109 bladder cancer pathology slides and 73 corresponding adjacent tumor tissue slides from Xiangya Hospital, The Second Affiliated Hospital of Guizhou Medical University, and the First People's Hospital of Kaili. All of the samples were collected following protocols approved by the ethics committees of the respective hospitals. Post-surgery, the samples were immediately frozen in liquid nitrogen and stored at -80 °C. They were then fixed 24 hours in 4% paraformaldehyde (Biosharp, Cat. #: 23265645) at 4 °C, embedded in paraffin, and sectioned at five microns. IHC staining was performed using standard protocols, incubating the tissue with a polyclonal rabbit anti-human TBX3 antibody at 1:400 (Abcam, Cat. #: ab99302) followed by color development using a DAB (3,3 -Diaminobenzidine tetrahydrochloride) substrate kit (Beyotime, Cat. #: A0308). Microscopy was employed for monitoring the staining process. Protein expression levels were calculated by multiplying the percentage of positive cells by the immunostaining intensity and were scored using a four-tier system: -, +, ++, +++. Specifically, the scoring for the percentage of positive cells was: 0 points for non-positive cells, 1 point for 1-30%, 2 points for 31-60%, 3 points for 61-80%, and 4 points for 81-100%. Staining intensity was scored as follows: 0 points for no positive staining, 1 point for weak staining, 2 points for medium staining, and 3 points for strong staining. The final scores, based on the above criteria, were interpreted as follows: 0 points indicated no expression, 1-3 points indicated low expression, 4-8 points indicated moderate expression, and 9-12 points indicated strong expression. In addition, a tissue microarray (TMA) of the Xiangya immune cohort was prepared and IHC staining was performed as reported in our prior study 34.

### Description of the TME features based on bulk RNA-seq

The Cancer-Immunity Cycle was evaluated by uploading TPM data to the TIP website (http://biocc.hrbmu.edu.cn/TIP/). Details regarding the analysis processes can be found in our prior study 37-39. Gene sets for 28 immune cell infiltrations were sourced from Charoentong *et al.*, and the levels of these cells were quantified using single-sample gene set enrichment analysis (ssGSEA) 40. In addition, 122 immunomodulators were also derived from the study by Charoentong *et al.* Further, we identified 22 Immune Checkpoint Inhibitors (ICI) genes from Auslander *et al.* 41, 18 genes for calculating the T cell-inflamed score (TIS) from Ayers *et al.* 42, and effector genes for various immune cells, including type 1 T helper cells, CD8^+^ T cells, dendritic cells, natural killer cells, and macrophages, from our prior research 34,38.

### Pathway enrichment analysis and molecular subtypes of BLCA

The empirical Bayesian method in the “limma” R package was utilized to identify differentially expressed genes (DEGs), with the criteria set at a fold change (FC) > 2 and a p value < 0.05. Gene Ontology (GO) and Kyoto Encyclopedia of Genes and Genomes (KEGG) datasets were obtained from the Molecular Signatures Database (MSigDB) (https://www.gsea-msigdb.org/gsea/index.jsp). In addition, we also performed Gene Set Enrichment Analysis (GSEA) based on genes ranked by their FC values. The ssGSEA algorithm was employed to quantify the activity of 12 pathways associated with BLCA 43.

Currently, seven molecular subtypes are widely used in the classification of bladder cancer, namely, University of North Carolina (UNC), MD Anderson Cancer Center (MDA), Cartes d'Identité des Tumeurs (CIT), Lund, TCGA, Baylor, and Consensus 43. “ConsensusMIBC” and “BLCAsubtyping” R packages were used to define patients into different molecular subtype. Because these subtypes overlap or differ across systems, patients were categorized into basal and luminal types based on the consensus of the European Association of Urology's Bladder Cancer Molecular Typing Group 43. The accuracy of TBX3 in predicting these molecular subtypes was assessed using receiver operating characteristic (ROC) curve analysis.

### BLCA cell lines

T24 and TCCSUP cell lines were obtained from Procell Life Science & Technology (Wuhan, China). Mouse BLCA cells (MB49) were kindly provided by Professor Chen Ke from Tongji Hospital. The cells were cultured in 1640 DMEM medium (BasalMedia, China) supplemented with 10% fetal bovine serum (Gibco, USA) and 1% penicillin-streptomycin (NCM Biotech, China), and they were maintained in a 37 °C incubator with 5% CO_2_. For generating TBX3 stable knockdown cell lines, lentiviral vectors containing TBX3-short hairpin RNA (sh-TBX3) and TBX3-cDNA (oe-TBX3) were purchased from Shanghai Genechem. The targeting sequences were as follows: sh-TBX3 #Human 1: GCATACCAGAATGATAAGATA; sh-TBX3 #Human 2: GCTGCTGATGACTGTCGTTAT; sh-TBX3 #Mouse 1: GAAACAGAATTCATCGCCGTT; sh-TBX3 #Mouse 2: GCGAATGTTCCCTCCGTTTAA. BLCA cell lines were transfected using HiPerFect according to the manufacturer's instructions. Effective knockdown or overexpression of TBX3 was confirmed by real-time quantitative RT-PCR (qRT-PCR) and western blotting (WB).

### qRT-PCR

Total RNA was extracted using the Steady Pure Universal RNA Extraction Kit (Accurate Biology, China), and cDNA was synthesized with the Evo M-MLV RT Premix (Accurate Biology, China). qRT-PCR reactions were performed with the SYBR® Green Premix Pro Taq HS qPCR Kit (Accurate Biology, China), following the manufacturer's instructions. *GAPDH* served as the housekeeping gene for normalization. Primers were designed and synthesized by Sangon Biotech (Shanghai, China).

### WB

BLCA cells were cultured in 6-well plates. Once the cell growth reached 90% confluence, the cells were collected and lysed with 100 μl of RIPA buffer and 1 μl of a protease inhibitor cocktail (both from NCM Biotech, China). The protein concentration was measured using a BCA protein assay kit (NCM Biotech, China). Denatured proteins were resolved using PAGE gel in MOPS buffer and transferred to a PVDF membrane via wet transfer. The membrane was blocked in 5% TBST solution for two h at 20-25 ℃, and then incubated with a primary antibody overnight at 4 °C. The next day, the membrane was washed three times with TBST and incubated with the appropriate secondary antibodies at room temperature for 1 hour. Finally, the proteins on the PVDF membrane were visualized using the ECL system (NCM Biotech). The primary antibodies used in this study are listed in Supplementary Table S1.

### Enzyme-linked immunosorbent assay (ELISA)

The supernatant from a BLCA culture was collected, and the TGFβ1 concentration was measured using a human ELISA kit (Proteintech, USA) following the manufacturer's instructions. The optical density (OD) was read at 450 nm with a correction wavelength of 630 nm. A four-parameter logistic curve was used to fit the standard curve, from which the final target antibody concentration was calculated.

### Single-cell RNA-sequencing (scRNA-seq)

scRNA with Xiangya samples was performed as in our prior study^34^, with modifications in sample number. We added two new samples in this study, for a total of five samples. Briefly, subcutaneous tumors from three mice in each group were dissociated to obtain single-cell suspensions of immune cells for scRNA-seq. The MobiCube High-throughput Single Cell 3' Transcriptome Set V2.1 (PN-S050200301) and MobiNova-100 microfluidic platform were used for this process. The single-cell suspensions were adjusted to an appropriate concentration (700-1200 cells/μL) and immediately loaded onto a chip for micro-droplet formation using the MobiNova-100. Reverse transcription, cDNA amplification, and DNA library construction were performed according to the protocol High-throughput sequencing was carried out using the PE-150 mode.

Mobivision was employed to process the raw data and generate the UMI matrix. Cells with fewer than 1000 UMIs or with over 20% of transcripts derived from mitochondria were considered low-quality and were discarded. All the downstream analyses were conducted using Seurat (v 3.0.1) in the R environment (version 4.1.3). Because a library was constructed for every sample, sample IDs were used to mitigate potential batch effects with Harmony (https://github.com/immunogenomics/harmony). After performing principal component analysis (PCA), the top 50 PCs were utilized for tSNE or UMAP analysis. Clusters identified by Seurat's FindClusters function were annotated with known markers as described in the Results. Heatmaps of selected genes were generated using scanpy (version 1.7.1).

### Dual luciferase reporter assay

The luciferase reporter vectors were acquired from Promega. DNA fragments of every promoter or enhancer identified by Chromatin immunoprecipitation (ChIP) were amplified via PCR and inserted into the pGL3-basic luciferase reporter vector (HonorGene, Changsha, China). HEK293T cells were co-transfected with a reporter vector and TBX3 vector using Lipo2000 (Invitrogen, Cat. #: 11668-019, USA). After transfection for 48 h, the transfected cells were lysed in culture dishes containing a lysis buffer, and the resulting lysates were centrifuged at maximum speed for 1 min in a microcentrifuge. Relative luciferase activity was determined using a chemiluminescence detector (Promega, GloMax 20/20, USA), and the transfection efficiencies were normalized according to the activity of Renilla.

### Chromatin immunoprecipitation

Following the manufacturer's instructions (Abcam-ab500), 5 × 10^6^ cells were collected and cross-linked with 1% formaldehyde at room temperature for 10 minutes. The reaction was quenched with glycine, and cells were washed with PBS and pelleted. The cells were then sonicated to shear the DNA. Every reaction was incubated with 5 μg of an anti-TBX3 antibody. The protein-DNA complexes were captured on protein A/G agarose beads and washed with buffers of varying salt concentrations. The DNA was then extracted and precipitated. Specific primers were used for qPCR. The primers are listed in Supplementary Table S1.

### TissueFAXS panoramic analysis

To evaluate the relationship between TBX3^+^ cells, malignant cells, CD8^+^ T cells, and fibroblasts, we used the TissueFAXS panoramic system (TissueGnostics, Austria) to detect the expression of these cells through multiple immunofluorescent stains in tumor tissue biopsies from the Xiangya BLCA TMA cohort. Specifically, primary and secondary antibodies were used to stain TBX3^+^ cells, CD8^+^ T cells, and α-SMA^+^ cells, while a CK19 antibody was used to stain tumor cells. Cell nuclei were stained with DAPI (Invitrogen, USA) as described by Makarevic et al. For spatial analysis, we quantified CD8^+^ T cells and α-SMA^+^ cells around TBX3^+^CK19^+^ cells according to distance gradients (0-25 µm, 25-50 µm, 50-100 µm, and 100-150 µm).

### Cytokine-array analysis

The Human Cytokine Antibody Array-Membrane (Abcam, Cat. # ab133997) was used to evaluate the changes in 42 cytokines between TBX3-overexpressing tumor cells and control cells. BLCA cell culture fluid was collected and centrifuged at 10,000 × g for 10 minutes. The supernatant was used for further analysis. The antibody arrays were removed from their packaging and placed into the wells of the incubation tray. Every tray was filled with 2 ml of blocking buffer and incubated for 30 minutes at room temperature. The blocking buffer was then aspirated, and every well was filled with 1 ml of the sample and incubated overnight at 4 °C. Following aspiration, the wells were washed with 2 ml of 1× Wash Buffer I, incubated for 5 minutes at room temperature, and this process was repeated three times. Subsequently, 2 ml of 1× Wash Buffer II was added to every well and incubated for 5 minutes at room temperature. Next, 1 ml of the prepared Biotinylated Antibody Cocktail was added to every well and incubated for 2 hours at room temperature, after which it was aspirated and the membranes were washed again. Then, 2 ml of 1× HRP-Streptavidin was added to every well and incubated for 2 hours at room temperature, followed by another washing step. The membranes were transferred onto chromatography paper. A mixture of equal volumes of Detection Buffer C and Detection Buffer D (500 µl each) was gently pipetted onto every membrane and incubated for 2 minutes at room temperature. Finally, the membranes were scanned using the e-BLOT imaging system (Touch Imager, China).

### Co-culture assay

Peripheral blood mononuclear cells (PBMCs) were isolated from the blood samples of healthy volunteers using density gradient centrifugation with Lymphoprep mononuclear cell separation fluid. The PBMCs were cultured in ImmunoCult T Cell Expansion Medium supplemented with 50 U/ml of IL-2 and ImmunoCult CD3/CD28/CD2 T cell Activator. The cells were maintained in this culture medium for three days before co-culturing. A Transwell chamber with a 0.4-μm pore size membrane was used for the co-culture assays. In the co-culture setup, 2.5 × 10^5^ TCCSUP tumor cells (either TBX3-overexpressing or TBX3-vector) were placed in the top insert of the Transwell and co-cultured with 5 × 10^5^ CD8^+^ T cells and 2.5 × 10^5^ HBdSF fibroblasts in the lower chamber. On the second day, 10 µg/ml of TGF-β1 neutralizing antibody (BioXcell, BE0057) or isotype IgG was added to the upper chamber. The culture medium was replaced once at the midpoint of the co-culture period. Co-culture with CD8^+^ T cells lasted for seven days, while co-culture with HBdSF fibroblasts lasted for five days. CD8^+^ T cells were analyzed using flow cytometry, and fibroblasts were examined using western blotting.

### Animal experiment

After obtaining approval from the appropriate animal ethics committee, C57BL/6 mice (6-8 weeks old) were purchased from Department of Laboratory Animals, Central South University. After one week of acclimatization, BLCA cells were inoculated into the lateral dorsal area of the mice. Cultured MB49 cells were digested with trypsin, washed twice with pre-cooled PBS, resuspended, and counted. The cell concentration was adjusted to 5 × 10^6^ cells/ml. Using an insulin syringe, 100 μl of cells were subcutaneously injected into the mice, ensuring no cell leakage. The injection and withdrawal of the needle were performed in a curved path. The mice were weighed, and tumor sizes were measured every three days. The study included four experimental protocols. Protocol 1 included MB49-vector and MB49-oeTBX3 groups. Protocol 2 included MB49-oeTBX3-shNC and MB49-oeTBX3-shTGFβ1 groups. Protocol 3: After successfully establishing the subcutaneous tumor model, the mice were administered nintedanib (30 mg/kg per mouse) or sterile ultrapure water by gavage daily for two weeks. Protocol 4: After successfully establishing the subcutaneous tumor model with shTBX3 and shNC MB49 cell lines, the mice in the different groups were intraperitoneally injected with 100 μg of αPD-1 or IgG2a isotype control. Treatments were administered every two days for a total of five times. After the treatment period, the mice were euthanized. Subcutaneous tumors were then isolated and washed with PBS, and tumor volumes were measured and photographed. The detailed grouping is as follows: shNC+IgG2a group, shTBX3+IgG2a group, shNC+αPD-1 group, and shTBX3+αPD-1 group. In addition, to assess the survival time of mice in every group, another set of mice was used to establish the model, extending the observation period to 60 days. The survival outcome was defined by either the subcutaneous tumor model exceeding 2000 mm^3^ or the death of the mice.

### Flow cytometry analysis

Tumor tissues from mice were digested into single-cell suspensions for flow cytometry analysis. Tubes containing 1 × 10^6^ cells were prepared, and PBS was added to every tube to reach a volume of 200 μl. The tubes were categorized into single-stain tubes, blank control tubes, and sample tubes. The blank control tubes contained only the cell suspension without any additional reagents. For the sample tubes, 1 μl of Fc receptor antibody was added for blocking and incubated at room temperature for 10 minutes. Subsequently, a viability dye was added to the sample tubes and incubated in the dark at room temperature for 10 minutes. Then, 2000 μl of PBS was added, and then the samples were mixed, and centrifuged at 400 × g for 5 minutes. The supernatant was discarded, leaving approximately 100 μl. Then primary antibodies were added to the sample tubes and incubated at 4 °C in the dark for 30 minutes. Permeabilization solution was prepared according to the manufacturer's instructions, and 300-500 μl of fixative was added and incubated at 4 °C in the dark for 50 minutes. Next, 2000 μl of 1× wash buffer was added. The samples were then centrifuged at 450 × g for 5 minutes, and the supernatant was discarded. Intracellular antibodies were added to the samples, followed by 50 μl of wash buffer. The samples were then incubated at room temperature for 30-50 minutes. Finally, 2000 μl of wash buffer was added to the samples, which were then centrifuged at 450 × g for 1 minute. The supernatant was discarded, and then the cells were resuspended in 200 μl of PBS and analyzed by flow cytometry.

### Statistical analysis

All of the data are presented as the mean ± SD. Statistical analyses and plots were performed using R software (version 4.1.3) and GraphPad Prism 10. For normally distributed data, comparisons between groups were made using Student's t test; otherwise, the Mann-Whitney U test was applied. For correlation analysis among continuous variables, a Pearson correlation coefficient was used for normally distributed data, while a Spearman correlation coefficient was used for non-normal data. A two-tailed p value < 0.05 was considered to be statistically significant.

## Results

### TBX3 expression pattern and potential role in the TME across different cancer types

Prior studies have revealed that TBX3 plays a crucial role in carcinogenesis and exhibits abnormal expression patterns in various cancers 23. Therefore, we first conducted a pan-cancer analysis using the TCGA database to validate these abnormal expression patterns across 33 different types of cancer. As shown in **Figure S1**, TBX3 was significantly overexpressed in BLCA, colon adenocarcinoma (COAD), esophageal carcinoma (ESCA), and rectum adenocarcinoma (READ). Conversely, it was significantly downregulated in cervical squamous cell carcinoma and endocervical adenocarcinoma (CESC), and head and neck squamous cell carcinoma (HNSC). After that, we focused on the associations between TBX3 and the TME immune status across multiple cancers. We assessed 122 immunomodulators, including MHC, receptors, chemokines, and immune stimulators, as reported by Charoentong *et al.* 40 and found that most of these immunomodulators were significantly negatively related to TBX3 expression in several cancers, particularly in BLCA (**Figure S2A**). Further, the exclusive association between TBX3 and the four most important immune checkpoints (PD-L1, PD-1, LAG3, and CTLA4) was particularly evident in BLCA (**Figure S2B-E**). These immune checkpoints have been reported to be associated with inflamed TMEs 44. Thus, we speculated that TBX3 induces an immunosuppressive microenvironment primarily in BLCA, and as such, we focused on the role of TBX3 in the TME of BLCA in the following research. As expected, further analysis revealed that TBX3 was negatively correlated with immune cell infiltration in BLCA, testicular germ cell tumors (TGCT), and thyroid carcinoma (THCA) (**Figure S2F**).

### TBX3 is mainly expressed on malignant epithelial cells and fibroblasts in the TME of BLCA

**Figure 1:**
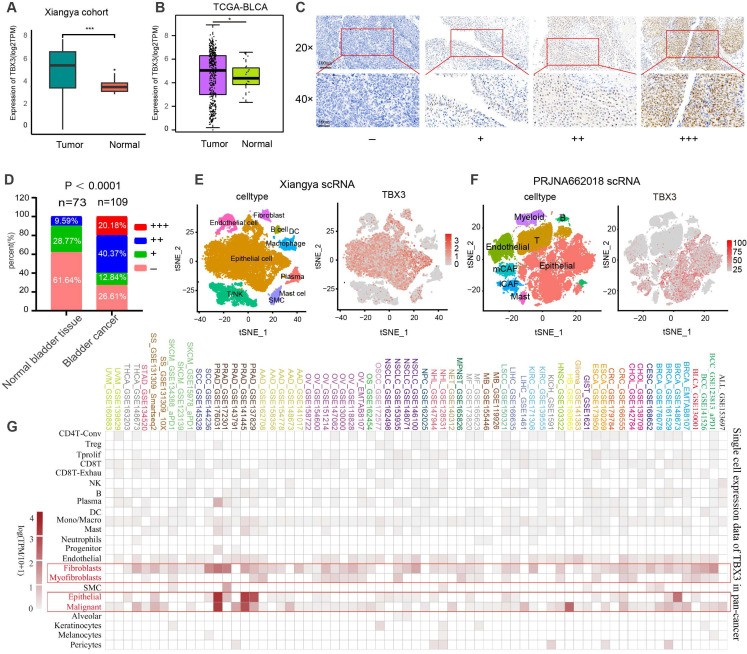
** TBX3 is highly expressed in bladder cancer, and it is mainly expressed in malignant epithelial cells and fibroblasts.** (A) Expression level of TBX3 in Xiangya bladder cancer cohort. (B) Expression level of TBX3 in the TCGA bladder cancer cohort. (C) The representative images of immunohistochemical staining showed the expression of TBX3 protein in bladder cancer and adjacent tissues. The yellow and brown nuclei represented positive. Scale bars, 100μm and 50μm. Expression levels of TBX3 were scored as four grades (-, +, + +, +++) by multiplying the percentage of positive cells and immunostaining intensity. (D)The positively stained nuclei (%) were analyzed by χ2 test. (E-F) The clustering of different cell types in Xiangya single cell cohort and PRJNA662018 cohort of bladder cancer tissue and the expression level of TBX3 in each cell type. (G)The heatmap shows the mRNA expression of TBX3 at the single-cell level across 33 tumor tissues. *P < 0.05, **P < 0.01, ***P < 0.001.

Based on bulk RNA-seq data, we found that TBX3 was expressed significantly higher in cancer tissues as compared to normal tissues in both the Xiangya cohort 34,38 (**Figure 1A**) and TCGA-BLCA cohort (**Figure 1B**). We further collected 109 BLCA cancer tissues and 73 para-carcinoma tissue, which were formalin-fixed and paraffin-embedded (FFPE,) from Xiangya Hospital, The Second Affiliated Hospital of Guizhou Medical University, and the First People's Hospital of Kaili. We performed IHC on these FFPE samples. As shown in **Figures 1C-D**, 60.55% of BLCA tissues showed moderate or high expression of TBX3, while this rate only reached to 9.56% in para-carcinoma tissue (p < 0.0001). Neither bulk RNA-seq nor IHC of FFPE samples are able to adequately assess the expression of TBX3 at the single-cell level in the TME. Therefore, as we previously reported^34^, we collected five BLCA samples for single-cell sequencing (named Xiangya scRNA) and found that TBX3 is primarily expressed in malignant epithelial cells and fibroblasts at the single-cell level (**Figures 1E-F**). The result was further validated using a public BLCA (PRJNA662018) scRNA-seq cohort 45. Further, this result also demonstrates extrapolative potential. We then analyzed the relative expression levels of TBX3 at the single-cell level across 33 types of cancer. This pan-cancer analysis indicated that TBX3 is primarily expressed in malignant tumor cells, epithelial cells, and fibroblasts across cancers (**Figure 1G**).

### TBX3 correlated with an immunosuppressive microenvironment in BLCA

**Figure 2:**
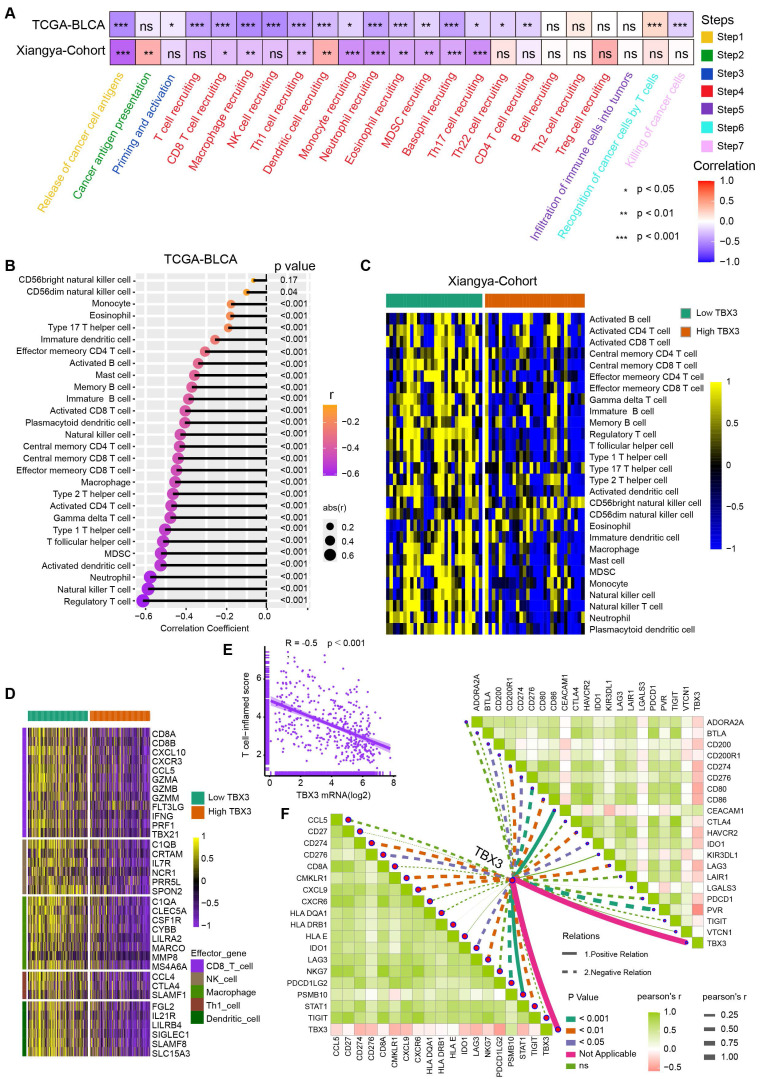
**TBX3 correlated with a non-inflammatory tumor microenvironment in BLCA.** (A) TBX3 expression and cancer immunity cycles in BLCA. The colors represent seven different steps. (B) Relationship between TBX3 and tumor-infiltrating immune cells (TIICs) by applying ssGSEA method within the TCGA-BLCA cohort. (C) Validation the interaction between TBX3 and TIICs in the Xiangya cohort. Taking the median expression level of tbx3 as the cut-off value, Xiangya cohort was divided into two groups. (D) Effector genes expression of CD8^+^ T cells, dendritic cells, NK cells, macrophages, and Th1 cells in high-TBX3 and low-TBX3 groups in TCGA-BLCA. (E) Correlation between TBX3 expression and T cell-inflamed scores in TCGA-BLCA. (F) Correlation between TBX3 expression and T cell-inflamed related genes (bottom left), and immune checkpoint genes (upper right) in the Xiangya cohort. *P < 0.05, **P < 0.01, ***P < 0.001.

Cancer-immunity cycle included seven immune response steps for immune surveillance 46. We found TBX3 was significantly negatively correlated with the majority of these steps both in the TCGA-BLCA and Xiangya cohorts (**Figure 2A**), indicating that TBX3 might induce an immunosuppressive microenvironment in BLCA through inhibiting the cancer-immunity cycle. Further, the infiltration of 28 types of immune cells were calculated using a ssGSEA algorithm. TBX3 was found to be significantly negatively correlated with the infiltration of effector memory CD4 T cells, activated CD8 T cells, effector memory CD8 T cells, and activated dendritic cells (**Figure 2B**). All of these cells have been shown to play vital roles in anti-tumor immunity. This result was validated in the Xiangya cohort (**Figure 2C**). Based on the median expression of TBX3 in the TCGA-BLCA cohort, we divided the TCGA-BLCA cohort into high and low TBX3-expressing groups and found that the immune effector genes from CD8^+^ T cells, natural killer (NK) cells, macrophages, type 1 T helper (Th1) cells, and dendritic cells were all expressed higher in the low TBX3-expressing group (**Figure 2D**). In addition, TBX3 was negatively related to T cell inflamed score (TIS) 42 (**Figure 2E**). Finally, TBX3 was negatively correlated with the component genes of TIS (**Figure 2F**, bottom left) and ICI genes (**Figure 2F**, upper right). All of these results were validated in GSE13507 (**Figure S3**) and GSE48075 (**Figure S4**). In summary, we found that TBX3 is related to an immunosuppressive microenvironment in BLCA.

### TBX3 inhibited CD8^+^ T cell accumulation and promoted CAFs infiltration

**Figure 3:**
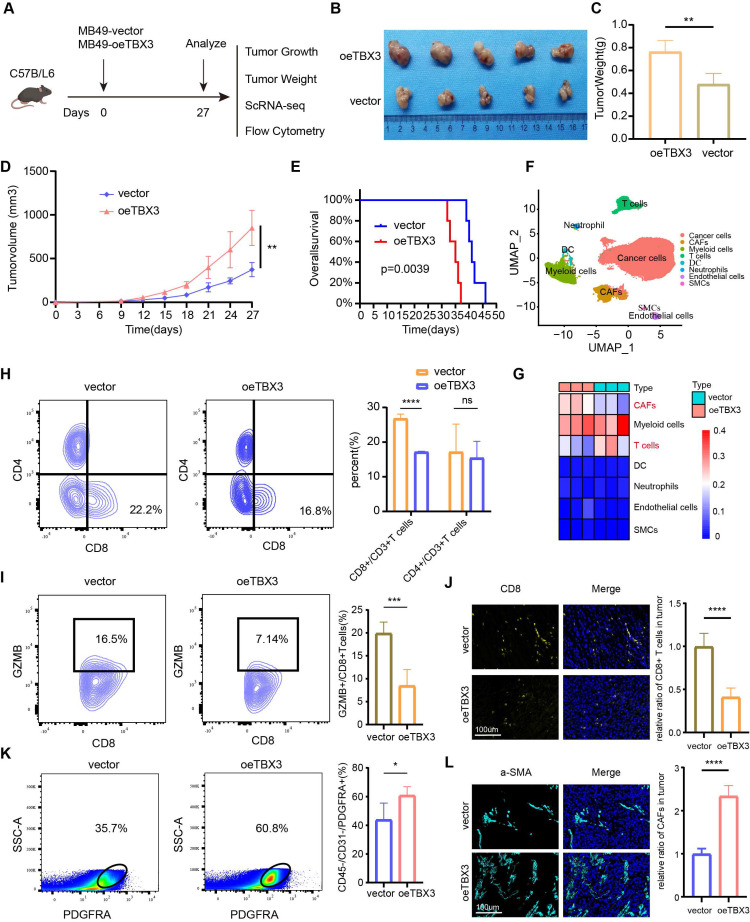
**TBX3 inhibited CD8^+^ T cell infiltration by promoting cancer associated fibroblasts**. (A) Flow chart of *in vivo* studies, created using the BioRender.com website. (B) Macroscopic view of subcutaneous tumor in C57BL/6 mouse. (C) Subcutaneous tumor weight of two group. (D) The volume curve of subcutaneous tumor in two groups of C57BL/ 6 mice. (E) Survival rates of both groups of mice. (F) Single-cell RNA sequencing of subcutaneous tumors (3 vs 3) in both groups of mice was clustered into seven major cell types. (G) The heat map shows the difference in the proportion of immune cell clusters between the two groups. (H) Flow cytometric analysis of CD8^+^ T cell, CD4^+^ T in subcutaneous tumors. (I) Flow cytometric analysis GZMB^+^ CD8^+^ T cell in subcutaneous tumors. (J) IF staining images of CD8^+^ T cell in subcutaneous tumors. (K-L) Flow cytometric analysis and IF staining images of CAFs in subcutaneous tumors. Mean ± SD, *P < 0.05, **P < 0.01, ***P < 0.001, ****P < 0.0001.

Our multi-omics analysis has already established a link between TBX3 and the immunosuppressive TME. We then investigated the mechanism underlying the formation of this immunosuppressive microenvironment *in vivo*. TBX3 overexpression (OE-TBX3) in human (TCCSUP) cells, TBX3 knockdown (shTBX3) in human (T24) cells, and murine (MB49) BLA cell lines were constructed successfully (**Figure S5**). A subcutaneous BLCA model was created by injecting MB49 cells with TBX3 overexpression or a negative control (**Figure 3A**). As shown in **Figures 3B-D**, TBX3 overexpression significantly promoted tumor growth in mice, indicating that TBX3 acts as an oncogene in BLCA. Further, TBX3 overexpression significantly decreased survival in mice (**Figure 3E**). We collected tumor tissues for scRNA-seq (**Figure 3F**), which revealed that TBX3 overexpression decreased the percentage of T cells while increasing CAFs (**Figure 3G**). Further, flow cytometry analysis showed that the affected T cell subtype was CD8^+^ T cells, not CD4^+^ T cells (**Figure 3H**). In addition, TBX3 overexpression inhibited GZMB expression on CD8^+^ T cells, suggesting that TBX3 reduces the cancer-killing efficiency of CD8^+^ T cells (**Figure 3I**). Flow cytometry analysis also confirmed that TBX3 overexpression promotes the infiltration of CAFs in the TME of BLCA (**Figure 3K**). These results were further validated by immunofluorescence staining (**Figures 3J-L**). In summary, TBX3 inhibited CD8^+^ T cell infiltration but promoted CAFs infiltration *in vivo*.

### TBX3 expression promoted the secretion of TGFβ1

**Figure 4:**
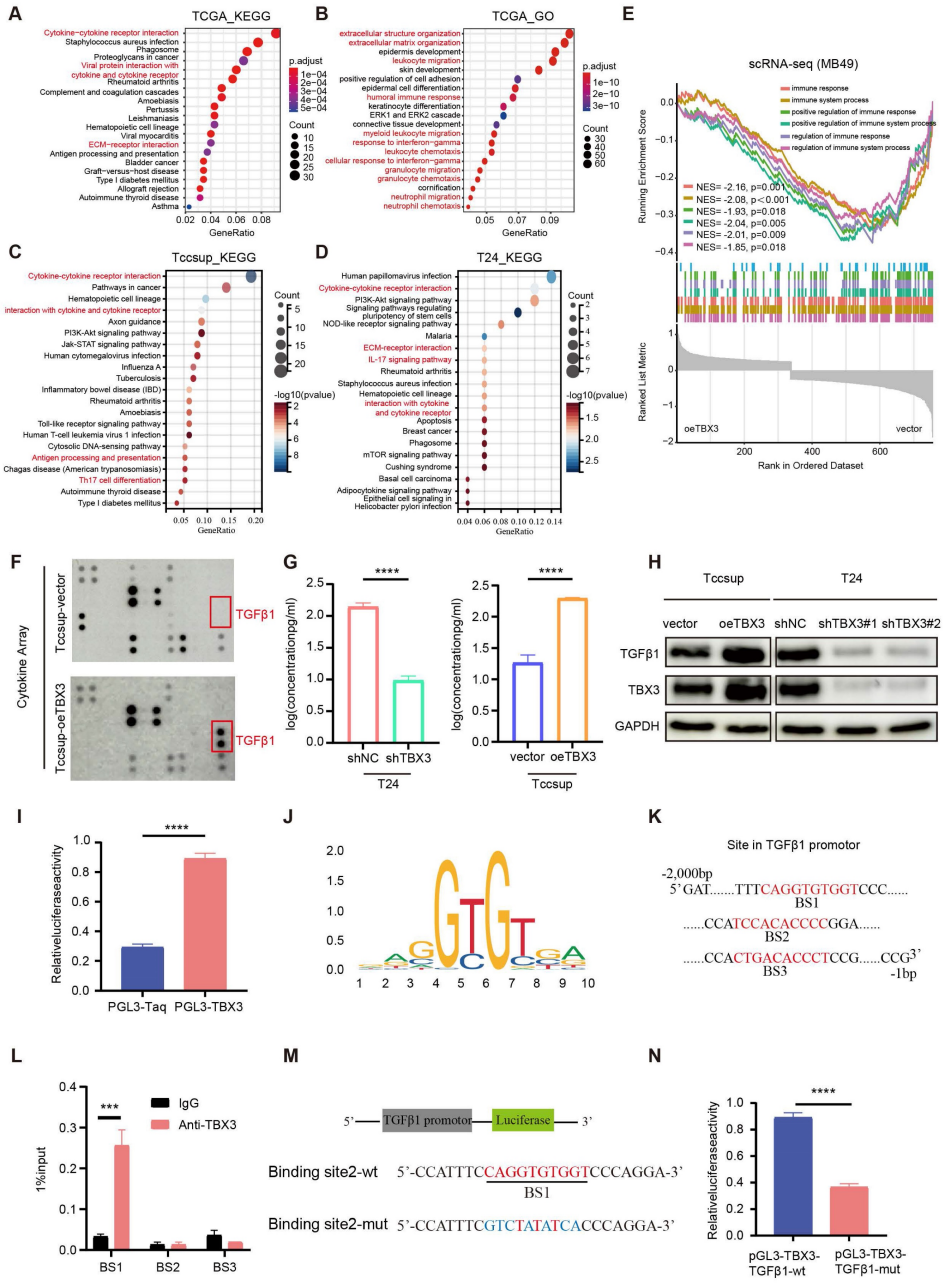
** TBX3 promotes the expression of TGFβ1 in bladder cancer cells.** (A, B) GO and KEGG enrichment analysis of differential genes in patients with high and low expression of TBX3 in TCGA bladder cancer cohort. (C) KEGG enrichment analysis of bladder cancer cells Tccsup-vector vs Tccsup-oeTBX3 differential gene. (D) KEGG enrichment analysis of bladder cancer cells T24-shNC vs T24-shTBX3 differential gene. (E) Differential gene GSEA enrichment analysis of cancer cells in subcutaneous tumors of MB49-vector and MB49-oeTBX3 groups. (F) Cytokine microarray analysis of overexpressed Tccsup-oeTBX3 and Tccsup-vector differences in expression of cytokines. (G) The levels of TGFβ1 secreted in bladder cancer cell lines were analyzed by Elisa. (H) The levels of TGFβ1 secreted in bladder cancer cell lines were analyzed by WB. (I) Luciferase reporter gene system analysis of TGFβ1 promoter activity in Tccsup cells co-transfected with PGL3-TBX3. (J) Transcription factor TBX3 binds to the motifs of downstream target genes. (K) Prediction of possible promoter binding sites of TBX3 and TGFβ1 by JASPAR database (top 3 scores). (L) qPCR-ChIP experiments confirmed that TBX3 directly binds to promoter TGFβ1. (M) Construct a mutation vector for binding site-1 luciferase reporter. (N) The activities of serially mutated tgfb1 promoter reporter vectors in the HEK293T cells co-transfected with pCMV-TBX3. Mean ± SD, p < 0.0001, p <0.0001.

To reveal the mechanism underlying TBX3 promoting an immunosuppressive microenvironment, we first performed GO and KEGG enrichment analysis based on the TCGA-BLCA cohort and found that the DEGs between the high and low TBX3 expression groups were mainly enriched on cytokine and extracellular matrix (ECM)-related pathways (**Figures 4A-B**). Further, we validated this result in TCCSUP and T24 BLCA cell lines (**Figures 4C-D**). The single cell data from **Figure 3F** further confirmed that overexpression of TBX3 can inhibit immune-activated pathways (**Figure 4E**). We used a high-throughput 42-cytokine membrane array to evaluate the changes in cytokine expression in the TBX3-overexpressing BLCA cell line TCCSUP in order to find the specific cytokines regulated by TBX3. We found significant changes in various cytokines in the TBX3-overexpressing BLCA cells, with TGFβ1 being the most notably altered (**Figure 4F**). Notably, TGFβ1 is the primary subtype of TGF in the TME and plays a crucial role in forming a non-inflamed TME. Prior studies have also indicated that TGFβ1 is both a key product and a major stimulant of CAFs, promoting the accumulation of CAFs and the deposition of the ECM 47,48. Therefore, we focused on TGFβ1 for further investigation. Upregulating TBX3 in BLCA cells increased TGFβ1 expression, while downregulating TBX3 decreased TGFβ1 expression (**Figures 4G-H**). Mechanistic studies revealed that TBX3 overexpression led to increased luciferase activity in the TGFβ1 promoter region (**Figure 4I**). In addition, the JASPAR database showed that the TBX3-binding motif in the target gene promoter includes GTGT (**Figure 4J**), and predicted the top three binding sites for TBX3 within the TGFβ1 promoter (**Figure 4K**). ChIP experiments demonstrated that TBX3 directly binds to binding site 1 of the TGFβ1 promoter (**Figure 4L**). By constructing a mutated TGFβ1 promoter reporter plasmid (**Figure 4M**), our study showed that mutation of binding site 1 reduced the activity of the TGFβ1 promoter, indicating the importance of binding site 1 for TBX3-mediated activation of the TGFβ1 promoter (**Figure 4N**). In summary, TBX3 promotes TGFβ1 expression by binding to its promoter.

### TBX3 promoted an immunosuppressive microenvironment through TGFβ1

**Figure 5:**
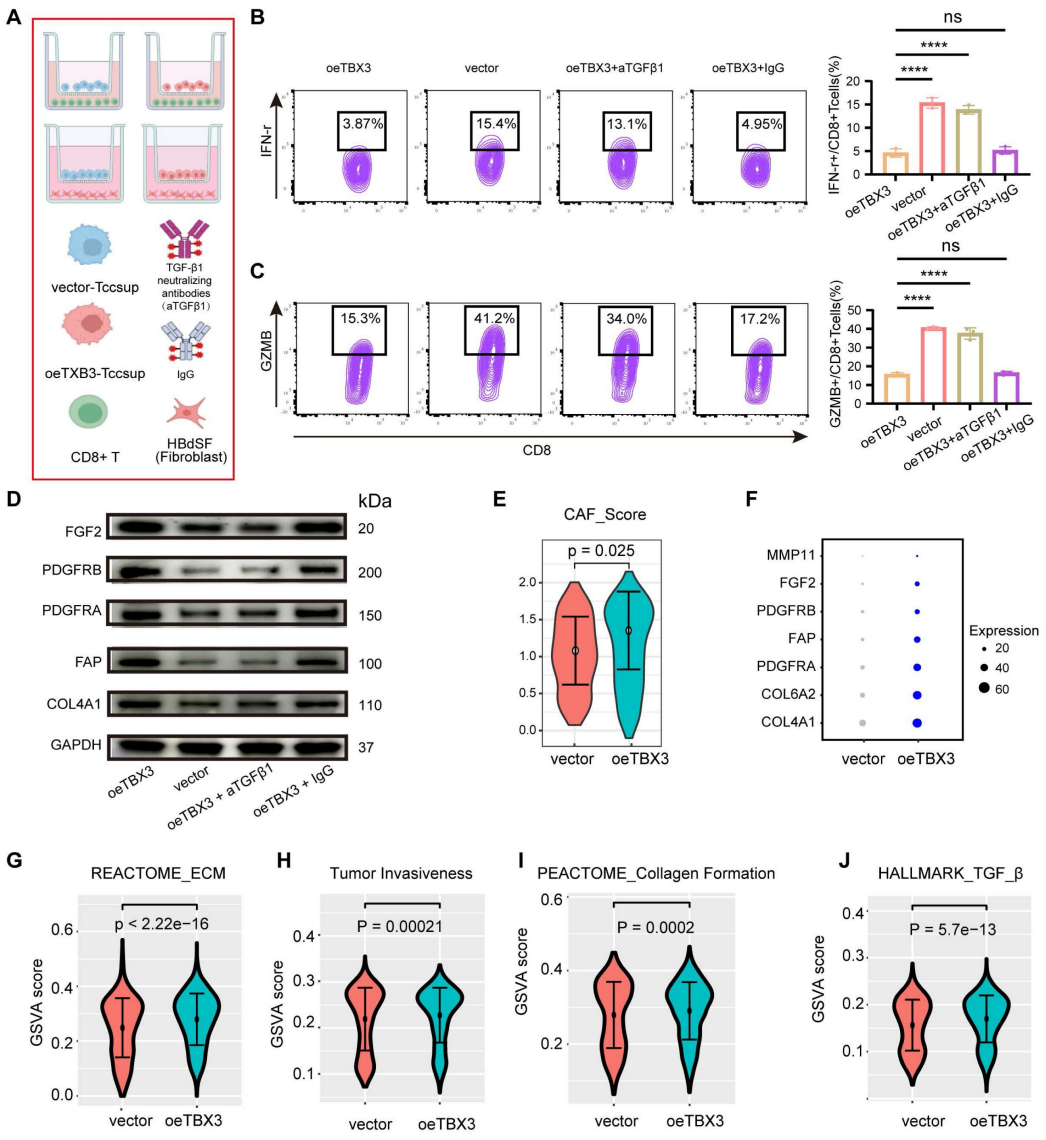
** High expression of TBX3 in tumor cells shapes the non-inflammatory tumor microenvironment in a TGFβ1-dependent manner.** (A) Co-culture workflow flowchart, created using the BioRender.com website. (B) Flow cytometry was used to analyze the proportion of IFN-r^+^ CD8^+^ T cells. (C) Flow cytometry was used to analyze the proportion of GZMB^+^CD8^+^ T cells. (D) Western blot analysis was performed on the labeled proteins of cancer-associated fibroblasts in the above four groups. (E) CAFs scores of cancer-associated fibroblasts in subcutaneous tumors in vector and oeTBX3 groups were compared. (F) The dot plot shows the average expression level of CAFs marker genes in vector and oeTBX3 groups. (G-J) Comparison of CAFs-related biological processes between vector and oeTBX3 groups.

We co-cultured TBX3-overexpressing BLCA cells with CD8^+^ T cells and fibroblasts in order to validate the key role of TGFβ1 in shaping an immunosuppressive TME (**Figure 5A**). Similarly, the results showed that co-culturing TBX3-overexpressing BLCA cells with CD8^+^ T cells significantly inhibited the cancer-killing efficiency of the CD8^+^ T cells (**Figures 5B-C**). Interestingly, when TGFβ1 was blocked with a TGFβ1-neutralizing antibody, the cancer-killing efficiency of CD8^+^ T cells significantly increased, along with an increased proportion of IFN-γ^+^ and GZMB^+^ CD8^+^ T cells (**Figures 5B-C**). Further, fibroblasts co-cultured with TBX3-overexpressing BLCA cells displayed higher levels of CAF markers, including FGF2, PDGFRB, PDGFRA, FAP, and COL4A1 (**Figures 5D-E**). As expected, this process was inhibited by the TGFβ1-neutralizing antibody (**Figure 5D**). The role of TBX3 in promoting CAFs was validated in scRNA-seq data. As shown in **Figures 5E-F**, the CAF score and markers were significantly higher in the TBX3-overexpression group 49. In addition, pathways related to ECM, tumor invasiveness, collagen formation, and TGF-β1 were all significantly activated in the TBX3-overexpression group (**Figures 5G-J**). We then knocked down TGFβ1 in the TBX3-overexpressing MB49 cell line and constructed a subcutaneous BLCA model (**Figure S6A**). We found that knockdown of TGFβ1 significantly inhibited the cancer-promoting ability of TBX3 (**Figures S6B-E**). Further, the knockdown of TGFβ1 also significantly inhibited the formation of an immunosuppressive microenvironment by increasing the percentage of CD8^+^ T cells (**Figure S6F**), GZMB^+^ CD8^+^ T cells (**Figure S6G**), and decreasing CAFs (**Figure S6H**) in the TME of BLCA. In summary, TBX3^high^ tumor cells promoted the infiltration of CAFs to form an immunosuppressive microenvironment through increased TGFβ1 secretion.

In addition, we explored the key role of CAFs in the formation of an immunosuppressive microenvironment. It has been reported that nintedanib can inhibit CAF infiltration 50, so we administered nintedanib to a subcutaneous BLCA model via gavage once daily for 14 days (**Figure S7A**). We found that the inhibition of CAFs significantly reduced the cancer-promoting ability of TBX3 (**Figures S7B-E**). The formation of an immunosuppressive microenvironment was also inhibited, as evidenced by lower levels of CAFs (**Figure S7F**) and a higher infiltration of CD8^+^ T cells (**Figure S7G**) and GZMB^+^ CD8^+^ T cells (**Figure S7H**).

### Downregulating TBX3 enhanced the efficiency of anti-PD-1 *in vivo*

**Figure 6:**
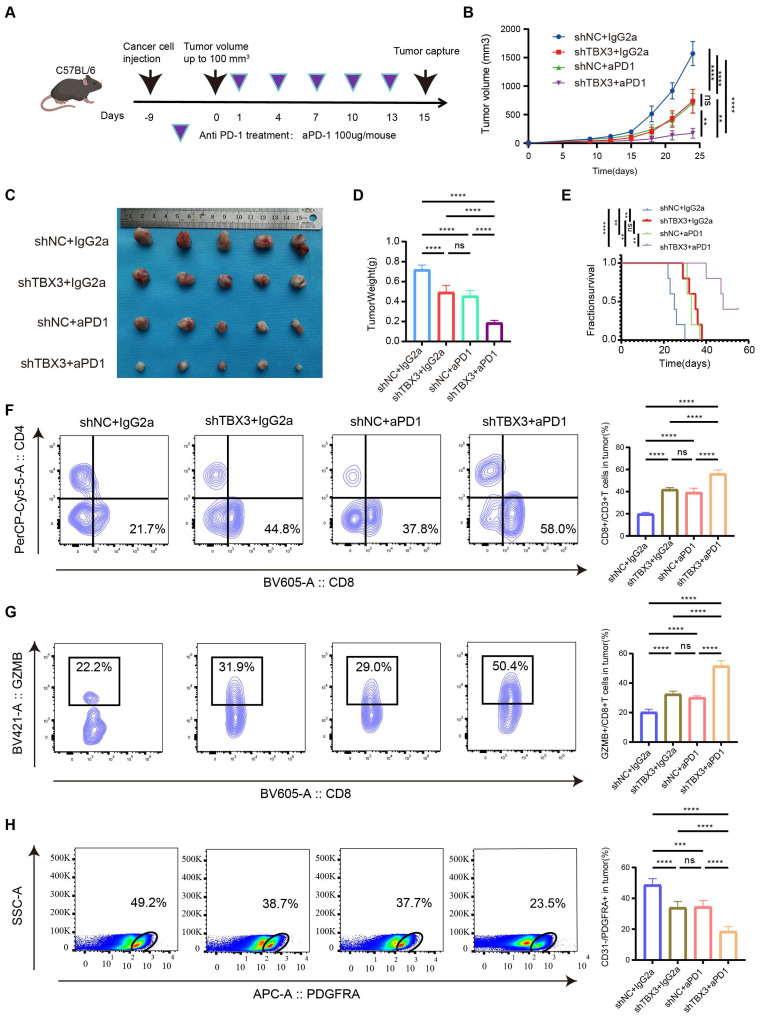
** Knocking down TBX3 inhibits tumor development and enhances anti-PD-1 therapeutic effect.** (A) Construction of mouse subcutaneous tumor model and flow chart of immunotherapy, created using the BioRender.com website. (B) Four groups of tumor growth volume maps. (C) Gross view of subcutaneous tumors in four group. (D) Changes of tumor weight of mouse in each group. (E) Survival curves of four groups of mice. (F) The proportion of CD8^+^ T in tumor tissue was analyzed by flow cytometry. (G) The proportion of GZMB^+^ CD8^+^ T cells in tumor tissue was analyzed by flow cytometry. (H) The proportion of CAFs in tumor tissue was analyzed by flow cytometry. *P < 0.05, **P < 0.01, ***P < 0.001, ****P < 0.0001.

A subcutaneous BLCA model was established using TBX3 knockdown or control MB49 cell lines, and the cells were treated with anti-PD-1 or IgG2a isotype antibody every three days for five cycles (**Figure 6A**). As expected, either downregulating TBX3 or anti-PD-1 treatment alone significantly inhibited the growth of BLCA and improved survival probability *in vivo* (**Figures 6B-E**). However, the most pronounced inhibition of cancer was observed in the group receiving a combination of TBX3 downregulation and anti-PD-1 treatment, indicating a synergistic effect (**Figures 6B-E**). Further, this combination group showed the highest infiltration of CD8^+^ T cells (**Figure 6F**) and GZMB^+^ CD8^+^ T cells (**Figure 6G**), along with the lowest levels of CAFs (**Figure 6H**). These findings suggested that the combination treatment reverses the immunosuppressive TME, thereby enhancing the efficacy of immunotherapy in BLCA.

### TBX3+ tumor cells were negatively associated with CD8+ T cell infiltration and positively associated with tumor-associated fibroblast aggregation in human BLCA

**Figure 7:**
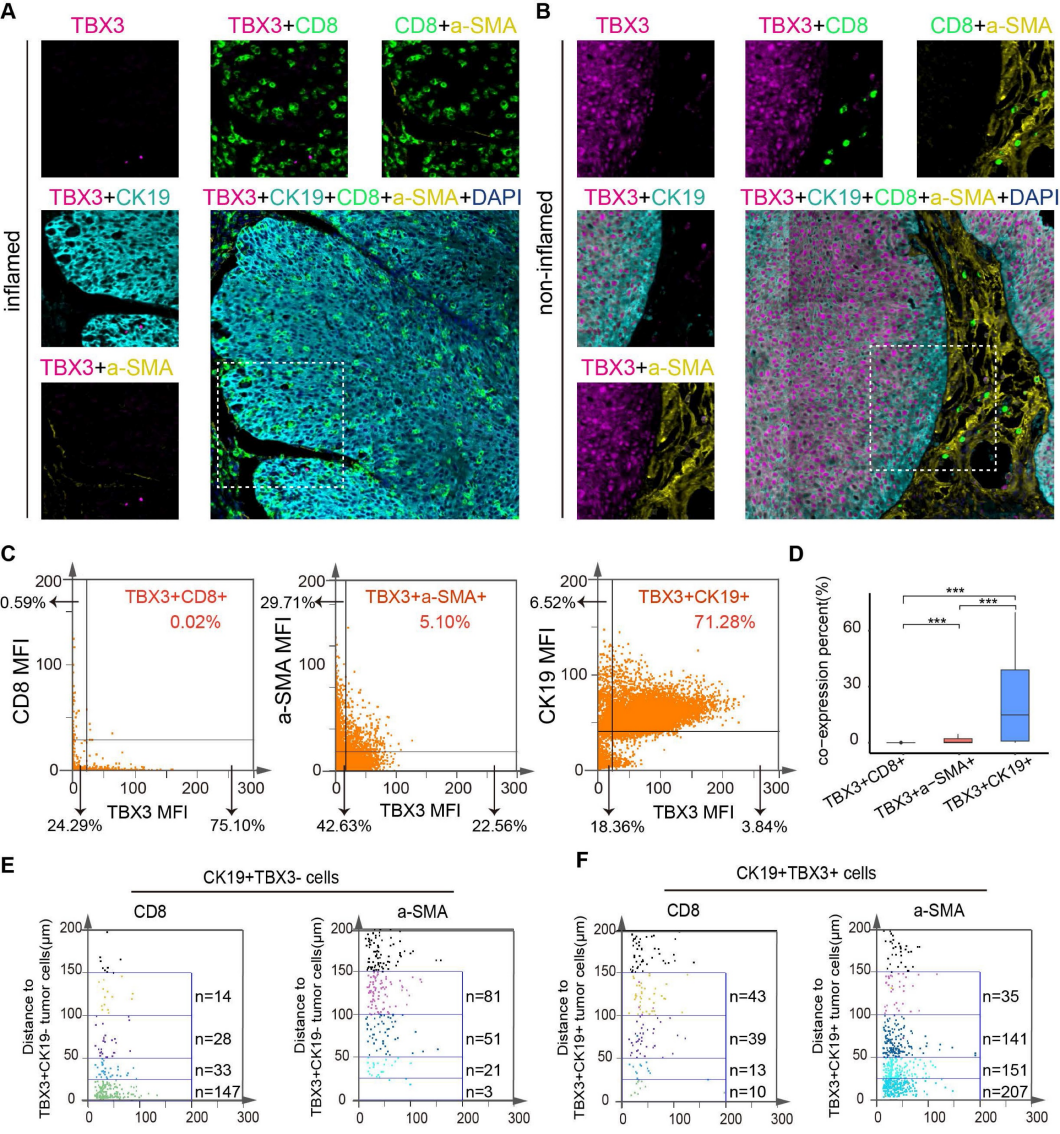
**TBX3^+^ tumor cells were negatively correlated with CD8^+^ T-cell infiltration and differentiation in human BLCA.** (A-B) Representative multicolor staining of inflamed (A) and non-inflamed (B) phenotypes of patients with BLCA in the Xiangya BLCA tissue microarray (TMA): TBX3(pink), CK19 (azure), CD8 (green), a-SMA (yellow), and DAPI (blue). (C) Representative flow cytometry-like plots of TBX3^+^ CD8^+^(left), TBX3+a-SMA+ (middle) and TBX3^+^ CK19^+^cells (right) in TMA, respectively. (D) The histograms of different TBX3^+^ CD8^+^, TBX3^+^ a-SMA^+^, and TBX3^+^ CK19^+^ percent cells among the whole TMA. (E-F) Gradient analysis for multidimensional distances (0-25µm, 25-50µm, 50-100µm, 100-150µm) showed the spatial distribution of TBX3^-^ tumor cells (E) and TBX3^+^ tumor cells (F) between CD8+cells and a-SMA+cells. ***P < 0.001.

In the above results, we demonstrated the immunosuppressive effect and potential mechanism of TBX3 in BLCA by analyzing the public databases, Xiangya bulk-RNA sequencing results, and Xiangya scRNA sequencing results, and performing an animal model of BLCA. However, in human tissue, the correlation between TBX3^+^ tumor cells and T cells and CAFs remains unclear. Therefore, we prepared a TMA containing 50 BLCA samples, namely, the Xiangya BLCA TMA, and performed multicolor staining on TBX3^+^ tumor cells (TBX3^+^ CK19^+^), CD8^+^ T cells, and CAFs (α-SMA^+^). The TissueFAXS panoramic quantitative platform was used for semi-automatic analysis to reveal the spatial relation of TBX3^+^ BLCA cells, CD8+ T cells, and CAFs. Tumors with low TBX3 expression exhibited inflammatory phenotypes, with a large number of CD8^+^ T cells infiltrating the tumor area, while tumor-associated fibroblasts infiltrated significantly less (**Figure** 7A). In contrast, high expression of TBX3 in tumor cells (CK19^+^) showed non-inflammatory phenotypes, and extensive expression of TBX3 in tumor cells resulted in a large accumulation of CAFs and inhibited CD8^+^ T cell infiltration in the tumor area (**Figure** 7B). Compared with the co-expression ratio of TBX3^+^ CD8^+^ T cells (0.02%) and TBX3^+^ α-SMA^+^ CAFs (1.02%), TBX3 was mainly expressed in BLCA cells (71.28%), which revealed similar results as the scRNA sequencing (**Figures** 7C**-**D). For further spatial analysis, we performed counts of CD8^+^ T cells and CAFs within the TBX3 expression gradients in tumor cells (CK19^+^) (0-25 µm, 25-50 µm, 50-100 µm, and 100-150 µm). As expected, as the distance from TBX3^+^ CK19^+^ cells increased, the number of CD8^+^ T cells gradually increased, while the CAFs decreased (Figure 7D**-**E). This result confirmed the spatially exclusive relationship between TBX3 and effector T cells, which may be the cause of immunotherapy resistance.

### TBX3 was associated with immunotherapy resistance in multiple real-world cohorts

**Figure 8:**
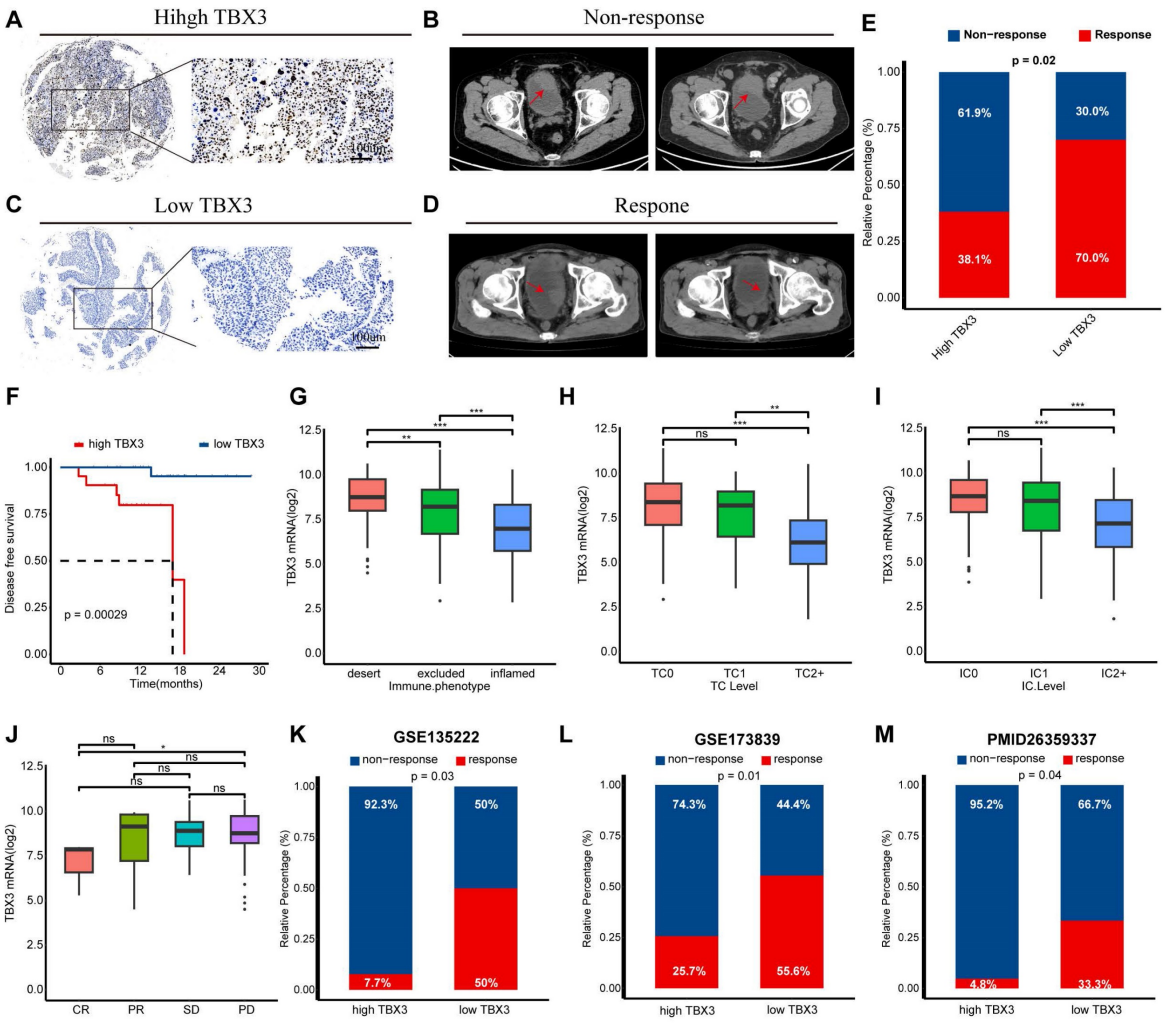
** Relationship between TBX3 expression and immune checkpoint blockade (ICB) response.** (A) Representative immunohistochemical (IHC) image of high TBX3 expression patient. Scale bar, 100 μm. (B) Representative CT image for patient with progressive disease after anti-PD-1 treatment. (C) Representative IHC image of low TBX3 expression patient. Scale bar, 100 μm. (D) Representative CT image for patient with complete response after anti-PD-1 treatment. (E) Relative percentage of patients with clinical response to immunotherapy between different TBX3 expression groups in Xiangya immune cohort. Red, immunotherapy response group; Blue, non-response group. (F) Disease-free survival (DFS) of patients with different TBX3 IHC scores in Xiangya immune cohort. (G) Expression of TBX3 on desert, excluded, and inflamed immune phenotypes in IMvigor210 cohort. (H-I) Expression of TBX3 on bladder cancer with different PD-L1 expression on immune cells (H) and tumor cells (I) in IMvigor210 cohort. (J) Correlation between TBX3 expression values and immunotherapy response in the desert phenotype of IMvigor210 cohort. Different color represents different response type. CR: complete response; PR: partial response; SD: stable disease; PD: progressive disease. (K-M) Relative percentage of patients with clinical response to immunotherapy between different TBX3 expression groups in GSE135222, GSE173839, PMID26359337 cohorts. Red, immunotherapy response group; Blue, non-response group. ns, not statistically significant. *p < 0.05; **p < 0.01; ***p < 0.001.

We have previously demonstrated that TBX3 promotes an immunosuppressive microenvironment and that downregulating TBX3 enhances the efficacy of anti-PD-1 therapy both *in vitro* and *in vivo*. We then aimed to determine the predictive value of TBX3 for immunotherapy efficacy in real-world cohorts. As reported in our prior study, we established a real-world BLCA immunotherapy cohort, named the Xiangya immune cohort 10,34. Our findings revealed that patients with high TBX3 expression were associated with immunotherapy resistance (**Figures 8A-B**), whereas patients with low TBX3 expression were sensitive to the treatment (**Figures 8C-D**). These results were statistically significant across the entire cohort (**Figure 8E**). In addition, patients with higher TBX3 expression exhibited significantly worse disease-free survival rates following treatment (**Figure 8F**). The IMvigor210 cohort, the largest BLCA immunotherapy cohort reported, indicated that TBX3 expression was significantly higher in patients with a desert immune phenotype (**Figure 8G**), low PD-L1 expression on tumor cells (TC0) (**Figure 8H**), and low PD-L1 expression on immune cells (IC0) (**Figure 8I**). Further, low TBX3 expression was associated with a complete response (CR) in the desert immune phenotype of the IMvigor210 cohort (**Figure 8J**). Lastly, we observed that TBX3 was associated with immunotherapy resistance in additional datasets, including GSE135222 51 (**Figure 8K**), GSE173839 52 (**Figure 8L**), and the cohort reported by Van *et al.* 36 (**Figure 8M**).

### Relationships between TBX3 and molecular subtypes of BLCA

BLCA is a heterogeneous disease that can be classified into different molecular subtypes based on gene expression profiles. These molecular subtypes can guide the treatment of BLCA and reflect distinct molecular characteristics 43. We categorized the TCGA-BLCA cohort into two groups based on the median expression of TBX3 and discovered that lower TBX3 expression was associated with the basal subtype of BLCA, which is known to be sensitive to immunotherapy and exhibits higher immune cell infiltration 43 (**Figure S8A**). The Area Under Curve (AUC) in **Figure S8B** demonstrated that TBX3 has relatively high predictive accuracy for molecular subtypes. In addition, the percentage of mutated patients was higher in the low TBX3 group (**Figure S8C**). Beyond immunotherapy, we also investigated the response to chemotherapy, targeted therapy, and radiotherapy in patients with varying TBX3 expression levels. The findings indicated that patients with lower TBX3 expression might be sensitive to immunotherapy, radiotherapy, chemotherapy, and Anti-HER2 (ErbB2) therapy. In contrast, patients with higher TBX3 expression appeared to be responsive only to antiangiogenic therapy (**Figures S8D-E**). The relationship between TBX3 and BLCA molecular subtypes was further validated in the Xiangya cohort, GSE13507, and GSE48075 datasets (**Figure S9**).

## Discussion

BLCA is one of the most prevalent carcinomas worldwide, imposing a significant threat to human health and a heavy burden on society and the economy 1. Particularly, MIBC, which has a high potential to metastasize to lymph nodes, lungs, bones, and livers, is associated with a very poor prognosis and is generally considered incurable 2,3,53. Due to its high TMB, BLCA patients show partial responsiveness to ICB-based immunotherapy, leading to notable progress in recent years 4,5. However, identifying patients who are sensitive to immunotherapy versus those who are resistant and developing immune combination treatments are vital for BLCA treatment. Although ICBs represented by anti-PD-1 /PD-L1 have been beneficial for tumor patients, the immunotherapy response rate in BLCA is only about 20% 6-9,54,55. Although PD-L1, TMB, tumor-infiltrating lymphocytes, neoantigen burden, and TIS scores are reported to have potential in predicting immunotherapy efficiency 56, these biomarkers face significant challenges. For example, about 5% of patients with low TMB are sensitive to immunotherapy, while more than half of patients with high TMB do not respond 57. PD-L1 expression assessment faces similar issues, with around 11% to 20% of patients with PD-L1-negative tumors showing an objective response. In addition, the variability in PD-L1 immunohistochemistry antibodies and positive thresholds greatly limit its clinical use 56. Currently, there is no reliable biomarker for predicting BLCA immunotherapy efficiency.

TBX3 is a member of the T-box transcription factor family, playing a crucial regulatory role in embryonic development and organ formation 23. The TBX3 protein contains a T-box domain required for DNA binding and transcriptional initiation, two repression domains, and an activation domain 58. Studies have shown that TBX3 has a complex dual transcriptional regulatory function. On one hand, TBX3 represses transcription through a conserved repressor domain in their C-terminal regions 59. For example, Tbx3 can interact directly with HDAC5 through two crucial motifs (585LFSYPYT591 and 604HRH606) to facilitate cell migration by downregulating E-cadherin expression in hepatocellular carcinoma 60. TBX3 can enhance the proliferation of human embryonic stem cells by inhibiting p14ARF and NF-κB, which are regulators of the NF-κB signaling pathway 61. On the other hand, TBX3 acts as a transcription activator to upregulate target gene expression. Studies have shown that the induction of TBX3 expression in mouse embryonic fibroblasts can rapidly induce the expression of pluripotency factors, such as Sox2, Oct4, and Klf4 62. TROY activates the PI3K/AKT/TBX3 signaling, which helps to maintain the pluripotency of liver CSCs by upregulating the expression of SOX2, NANOG, and OCT4, and promotes cell motility via activating the EMT pathway in HCC 63. It is worth noting that TBX3 plays an irreplaceable role in the occurrence and development of cancer. For example, TBX3 is highly expressed in mammary tissues, and its overexpression is associated with the occurrence and progression of breast cancer. Studies have shown that TBX3 can promote the proliferation, migration, and invasion of breast cancer cells through various mechanisms while inhibiting apoptosis 64. Further, TBX3 interacts with androgen receptors, playing a key role in the progression and treatment resistance of prostate cancer 65. In addition, TBX3 can influence the development of head and neck squamous cell carcinoma by regulating the PTEN tumor-related signaling pathway 66. In addition to its carcinogenic role, TBX3 has also been shown to function as a tumor suppressor in fibrosarcoma and liver cancer, suggesting that the role of TBX3 is cancer-specific 67,68. Although there are some studies indicating TBX3 is an oncogene in BLCA 31,32, the role of TBX3 in the TME and tumor progression is not fully understood. In this study, for the first time, we focused on TBX3 in the TME of BLCA through pan-cancer analysis and revealed its key role in BLCA using multi-omics analysis and *in vitro* and *in vivo* experiments.

The malignancy and progression of tumors are influenced not only by the inherent invasiveness of cancer cells, but also by alterations in the TME, a complex system comprising activated fibroblasts, endothelial cells, pericytes, adipocytes, immune cells, and a rich ECM 69,70. Among these components, CAFs are particularly significant. CAFs interact with cancer cells and other cell types within the TME, playing a pivotal role in regulating tumor progression and therapeutic resistance 71. Notably, the interaction between CAFs and immune cells is crucial, with substantial evidence indicating that CAFs can suppress anti-tumor immunity through interactions with immune effector cells, particularly CD8^+^ T cells 72. For example, in pancreatic ductal adenocarcinoma, CAFs inhibit CD8^+^ T cell infiltration by secreting CXCL12 73. In addition, CAFs can modify the ECM to create physical barriers that restrict T cell movement 74. They also reduce CD8^+^ T cell recruitment by releasing IL-6 and TGF-β, inhibiting their cytotoxic activity against tumor cells 75,76. However, the mechanisms by which tumor cells mediate CAFs are less understood. In our study, overexpression of TBX3 significantly enhanced the promoter activity of the TGFβ1 gene and promoted TGFβ1 expression. Similarly, overexpression of TBX3 significantly enhanced the promoter activity of the TGFβ1 gene and promoted TGFβ1 expression. TBX3 has been shown to directly promote the expression of target genes by binding to specific DNA sequences within those genes. For example, Xiang Shi *et al.* found an enrichment of the GTGT motif in the promoters of human TAC3 and KISS1 genes and a robust stimulatory action of hTBX3 on these promoters, which promotes the expression of TAC3 and KISS1 77. In addition, Tbx3 directly activates Gata6 expression via its DNA binding activity. TBX3 has been shown to directly bind to the promoter of the Gata6 gene, a key regulatory protein of primitive endoderm differentiation, activating the expression of this gene 78. We discovered that TBX3^high^ tumor cells increase the secretion of TGFβ1, promoting CAF infiltration and creating an immunosuppressive microenvironment by inhibiting CD8^+^ T cell infiltration and their cancer-killing efficiency. Consequently, TBX3 is associated with resistance to immunotherapy. These findings are vital for BLCA, as TBX3 can serve not only as a biomarker for immunotherapy predicting, but also as a new target for immune combination treatment.

Our study has several limitations. First, our validation cohorts included RNA-seq and microarray data, which may have introduced batch effects and biases. Secondly, although we established that TBX3 promotes BLCA progression by inducing an immunosuppressive microenvironment, further research is needed to understand why tumor cells highly express TBX3. Finally, with the rapid development of nanomaterials, increasing nanomedicine delivery systems have been developed for the treatment of tumors 79-83. We will design TBX3 small-molecule nanodrug carrier inhibitors as the focus of future research.

## Conclusions

TBX3 promotes BLCA progression by inducing an immunosuppressive microenvironment, and targeting TBX3 could enhance the efficacy of immunotherapy for BLCA in the future.

## Supplementary Material

Supplementary figures and table.

## Abbreviations

TME: tumor immune microenvironment
BLCA: bladder cancer
TCGA: The Cancer Genome Atlas
GEO: Gene Expression Omnibus
RNA-seq: RNA-sequencing
FPKM: Fragments Per Kilobase of exon model per Million mapped fragments
TBX3: T-Box Transcript
TGFβ1: Transforming growth factor β1
CAFs: Cancer-associated fibroblasts
NMIBC: muscle-invasive bladder cancer
MIBC: muscle-invasive bladder cancer
TMB: tumor mutation burden
ECM: extracellular matrix
EMT: epithelial-mesenchymal transition
TMA: tissue microarray
IHC: Immunohistochemistry
TPM: Transcripts Per Kilobase per Million mapped reads
TIS: T cell-inflamed score
DEGs: differentially expressed genes
GO: Gene Ontology
KEGG: Kyoto Encyclopedia of Genes and Genomes
MSigDB: Molecular Signatures Database
GSEA: Gene Set Enrichment Analysis
ELISA: Enzyme-linked immunosorbent assay
scRNA-seq: Single-cell RNA-sequencing
COAD: colon adenocarcinoma
ESCA: esophageal carcinoma
READ: rectum adenocarcinoma
CESC: carcinoma and endocervical adenocarcinoma
HNSC: neck squamous cell carcinoma
TGCT: testicular germ cell tumors
THCA: thyroid carcinoma
FFPE: formalin fixation and paraffin embedding
