# Supplementary material for: TBX3 shapes an immunosuppressive microenvironment and induces immunotherapy resistance
Source: Theranostics. 2025 Jan 6;15(5):1966–86. doi: 10.7150/thno.103175 (PMC11780534; doi:10.7150/thno.103175)
Supplement: Supplementary file 1 — Supplementary figures and table. [file thnov15p1966s1.pdf]

Supplementary files

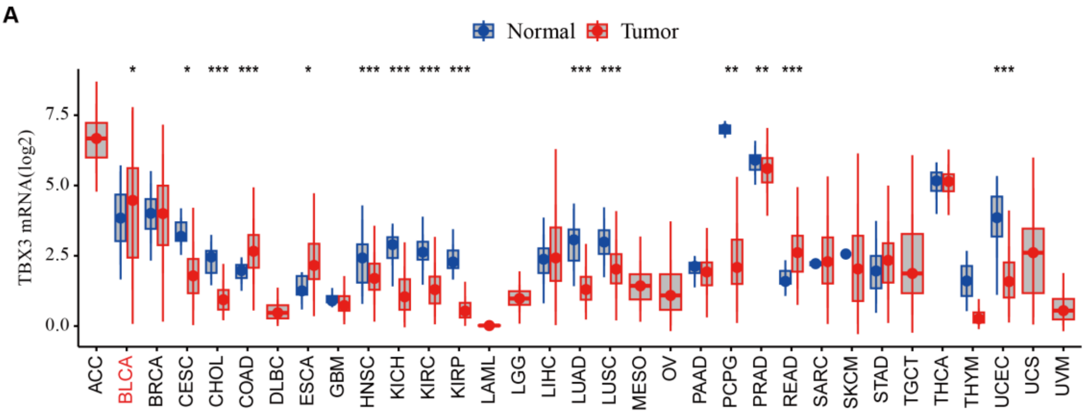

Figure S1 The mRNA expression of TBX3 in human pan-cancer.

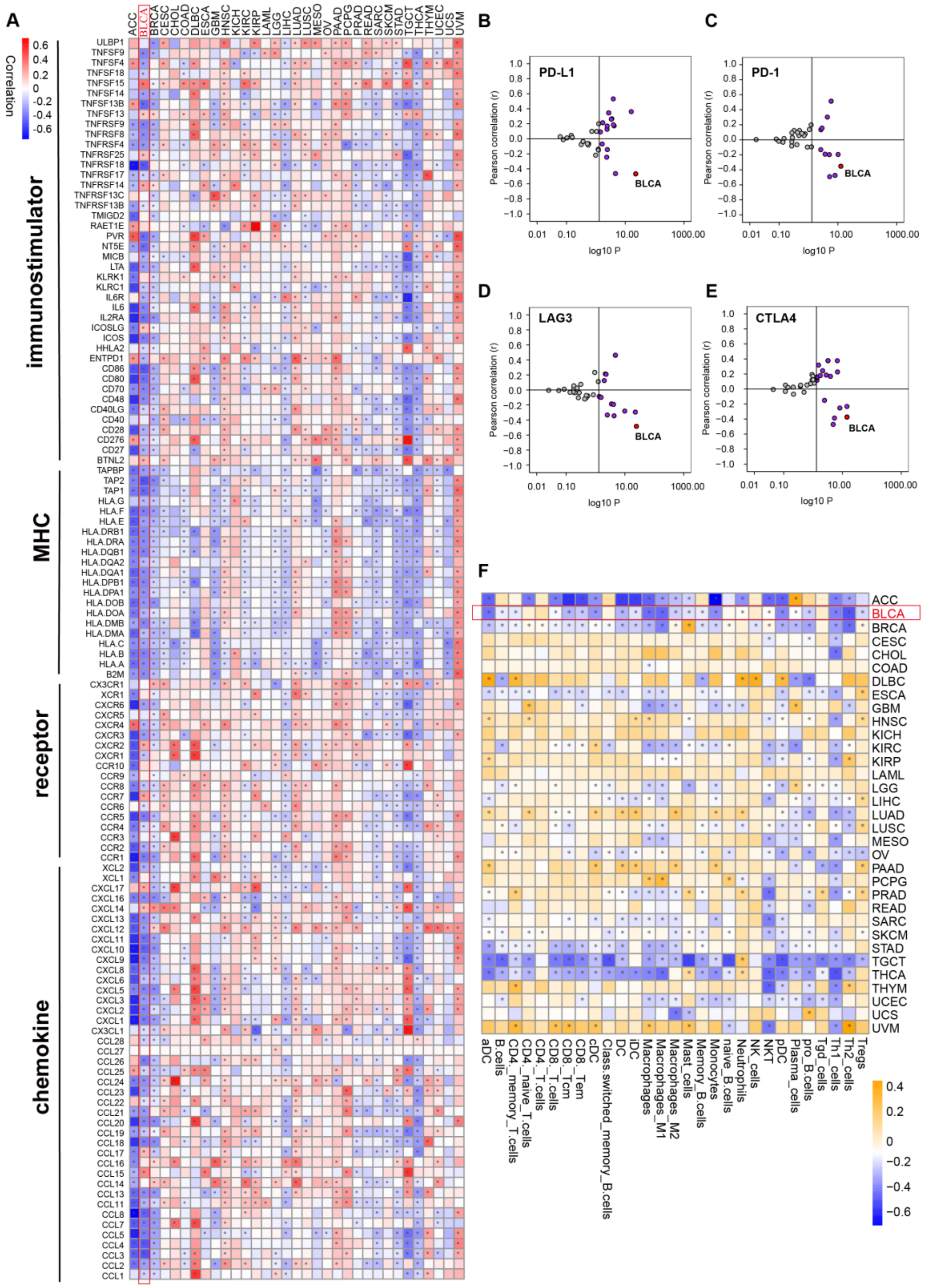

Figure S2. Correlation analysis of TBX3 and immune microenvironment characteristics in pan-cancer. (A) Heatmap of correlation between TBX3 and 122 immunomodulators in pan-cancer. (B-E) Correlation between TBX3 and four immune checkpoint molecules in pan-cancer. (F) Association between TBX3 and 28 tumor-infiltration-associated immune cells in pan-cancer.

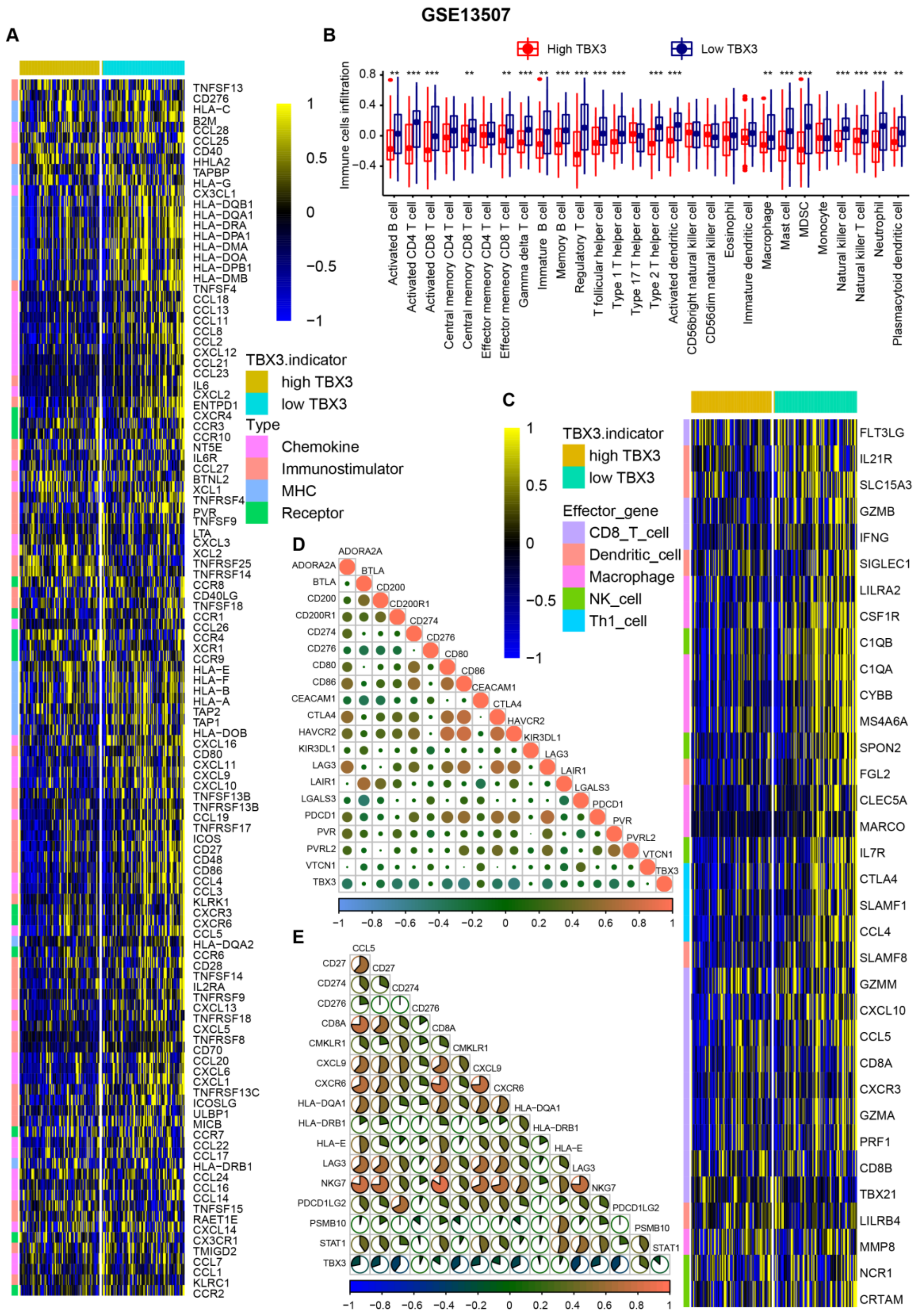

Figure S3. Characterizing the relationship between TBX3 and the non-inflammatory tumor microenvironment in the GSE13507 bladder cancer cohort. (A) Comparison of expression levels of immunoinflammatory regulators in High TBX3 group and Low TBX3 group. (B) The infiltration degree of inflammatory cells

was compared between the High TBX3 group and the Low TBX3 group. (C) The effector gene expression levels of five kinds of immune infiltration cells in the High TBX3 group and the Low TBX3 group were compared. (D) The relationship between TBX3 and immune checkpoint genes. (E) The relationship between TBX3 and TIS score effect gene.

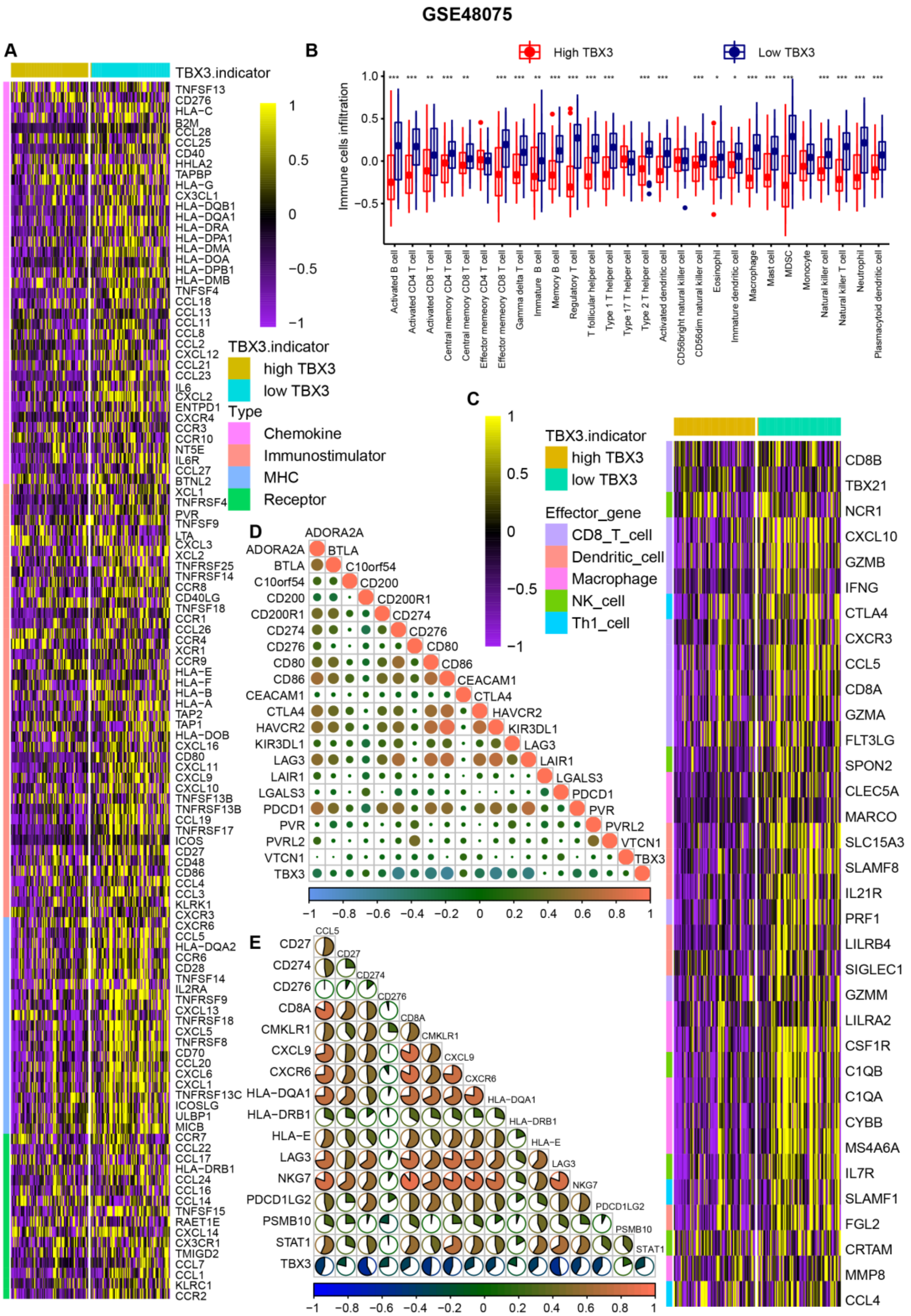

Figure S4. Characterizing the relationship between TBX3 and the non-inflammatory tumor microenvironment in the GSE48075 bladder cancer cohort. (A) Comparison of expression levels of immunoinflammatory regulators in High TBX3 group and Low TBX3 group. (B) The infiltration degree of inflammatory cells

was compared between the High TBX3 group and the Low TBX3 group. (C) The effector gene expression levels of five kinds of immune infiltration cells in the High TBX3 group and the Low TBX3 group were compared. (D) The relationship between TBX3 and immune checkpoint genes. (E) The relationship between TBX3 and TIS score effect gene.

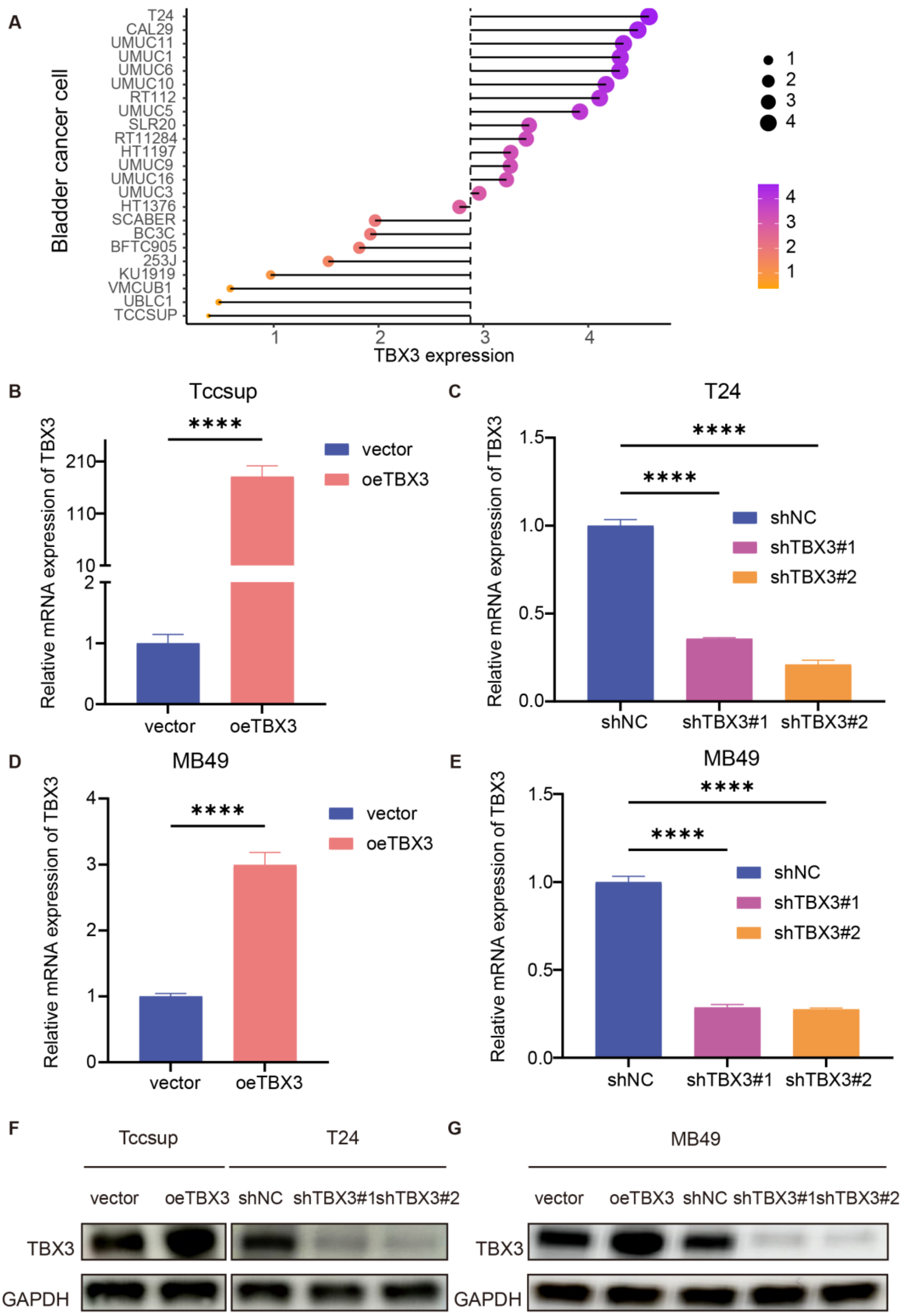

Figure S5. Construct TBX3 stable transmissible cell line. (A) Relative expression level of bladder cancer cell line TBX3. (B-E) qPCR was used to detect TBX3 expression which were overexpressed or knocked down in Tccsup, T24 and MB49 cells. (F) Western blot verified the successful construction of the above stable cells.

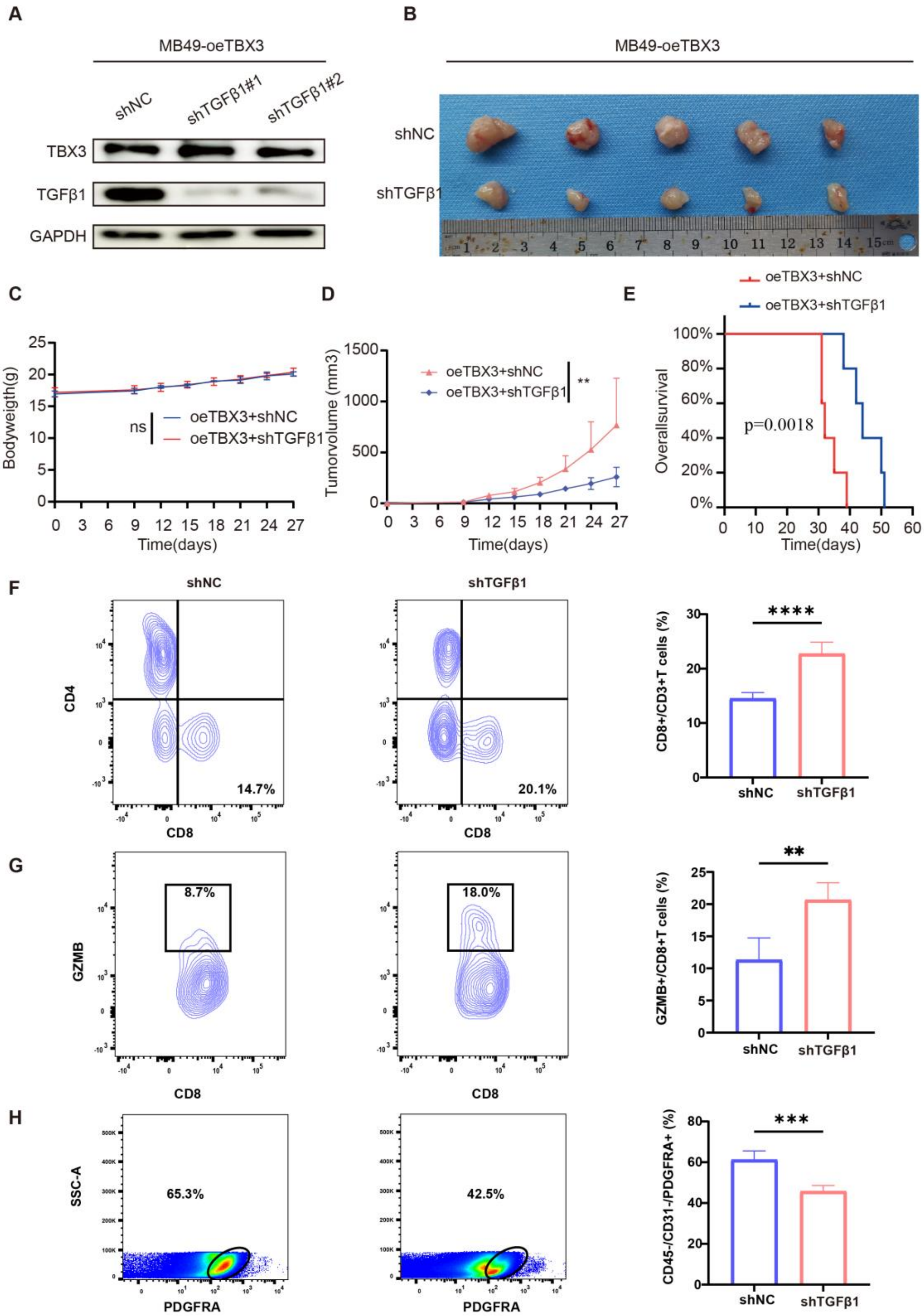

Figure S6. TBX3 promotes bladder progression through TGFβ1. (A) Western blot confirmed successful knockdown of TGFβ1 in MB49-TBX3 stable cell line. (B) Tumor image. (C) Weight changes of mouse in two groups. (D)The change curve of subcutaneous tumor volume in two groups of C57BL/6 mouse. (E) Survival time curve of two groups of mice. (F) The proportion of $CD8^{+}$T cells, $GZMB^{+}CD8^{+}$T cells

and CAFs in subcutaneous tumors of mouse in both groups was examined by flow cytometry.

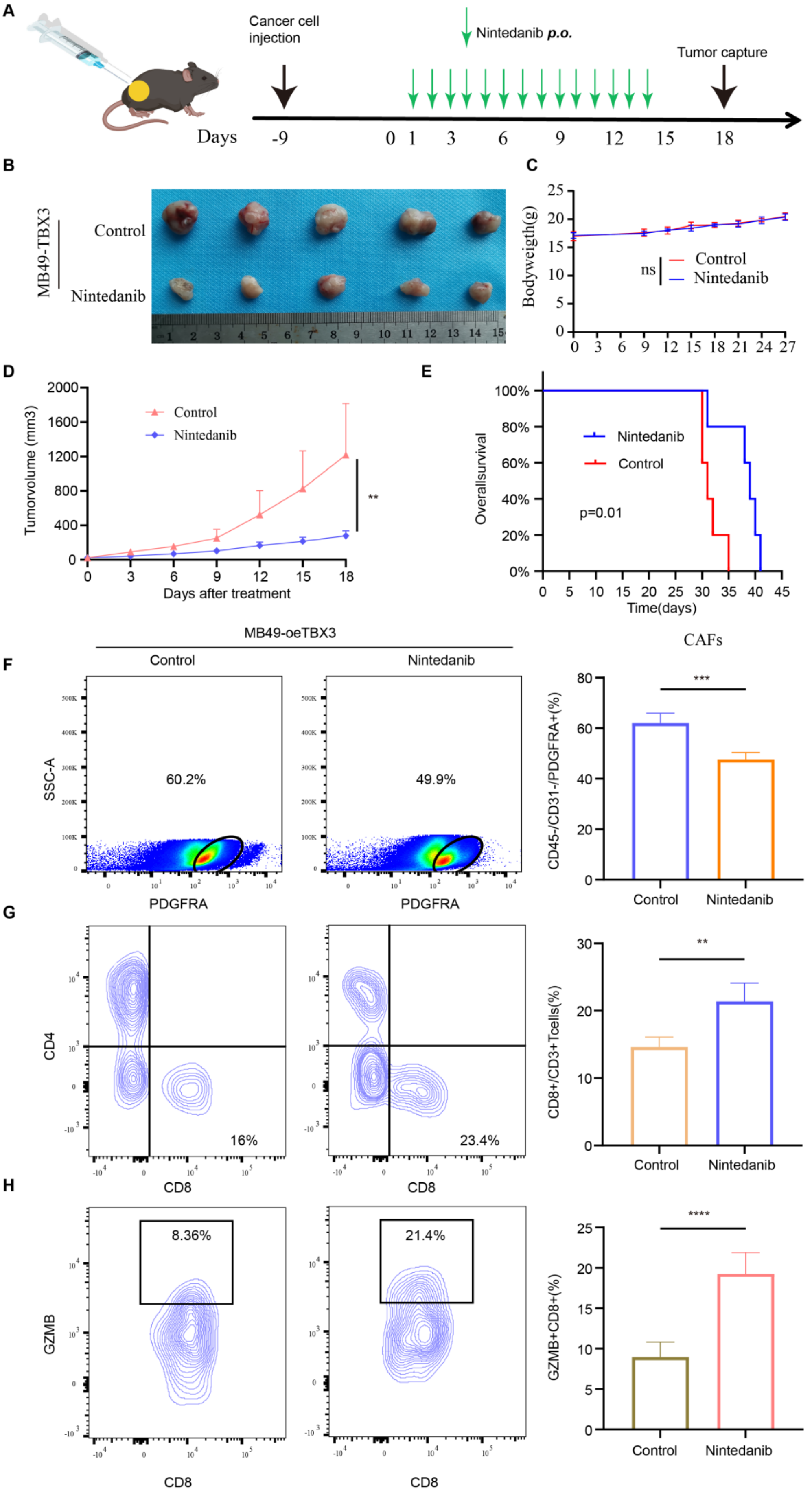
A
Cancer cell injection
Nintedanib p.o.
Tumor capture
Days -9 0 1 3 6 9 12 15 18
B
MB49-TBX3
Control
Nintedanib
C
Bodyweigth(g)
ns
Control
Nintedanib
D
Tumorvolume (mm3)
Control
Nintedanib
**
Days after treatment
E
Overallsurvival
Nintedanib
Control
p=0.01
Time(days)
MB49-oeTBX3
Control
Nintedanib
F
SSC-A
60.2%
49.9%
PDGFRA
CAFs
CD45-/CD31-/PDGFRA+(%)
***
G
CD4
16%
23.4%
CD8
CD8+/CD3+Tcells(%)
**
H
GZMB
8.36%
21.4%
CD8
GZMB+CD8+(%)
****
Control
Nintedanib

1

Figure S7. Antagonistic CAFs can inhibit the progression of TBX3-induced bladder cancer. (A) Subcutaneous tumor model construction and drug administration flow chart. (B) Direct view of subcutaneous tumor in two groups of mice. (C) Weight change curve of two groups of mice. (D) The change curve of subcutaneous tumor volume in two groups of C57BL/6 mouse. (E) Survival time curve of two groups of mice. (F) The proportion of $CD8^{+}$T cells, $GZMB^{+}$ $CD8^{+}$T cells and CAFs in subcutaneous tumors of mouse in both groups was examined by flow cytometry.

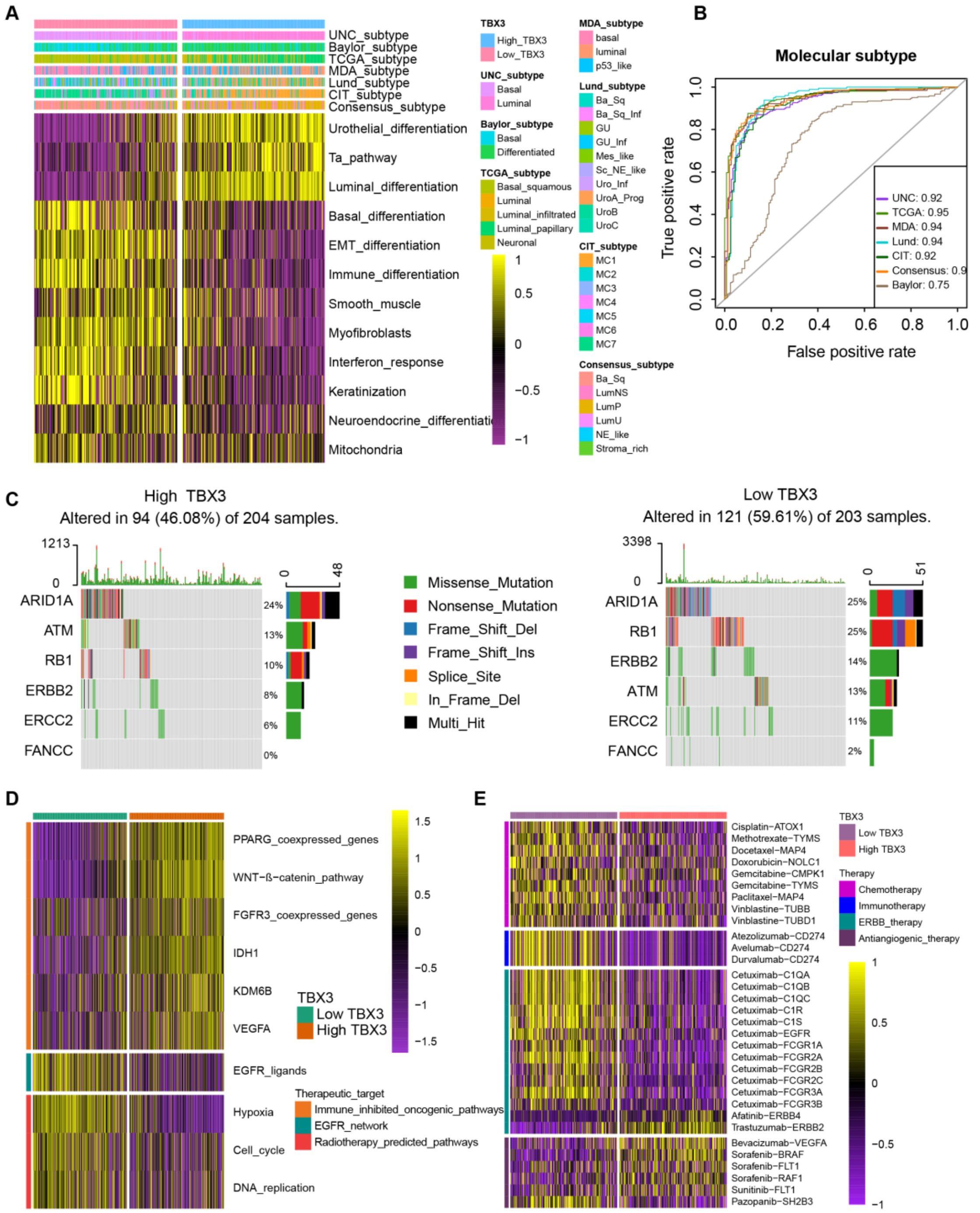

Figure S8. TBX3 predicts molecular typing and guides treatment in the TCGA bladder cancer cohort. (A) TBX3 high and low expression of 7 molecular typing results in two groups of patients, and the relationship with the activity of 12 gene sets. (B) ROC curve evaluated TBX3's accuracy in predicting 7 molecular typing. (C) Relationship between TBX3 and gene mutation associated with neoadjuvant chemotherapy. (D) Relationship between high and low expression of TBX3 and pathway activity of bladder cancer treatment regimen in two groups. (E) Relationship between high and low expression of TBX3 and common drug target genes in two

groups of patients.

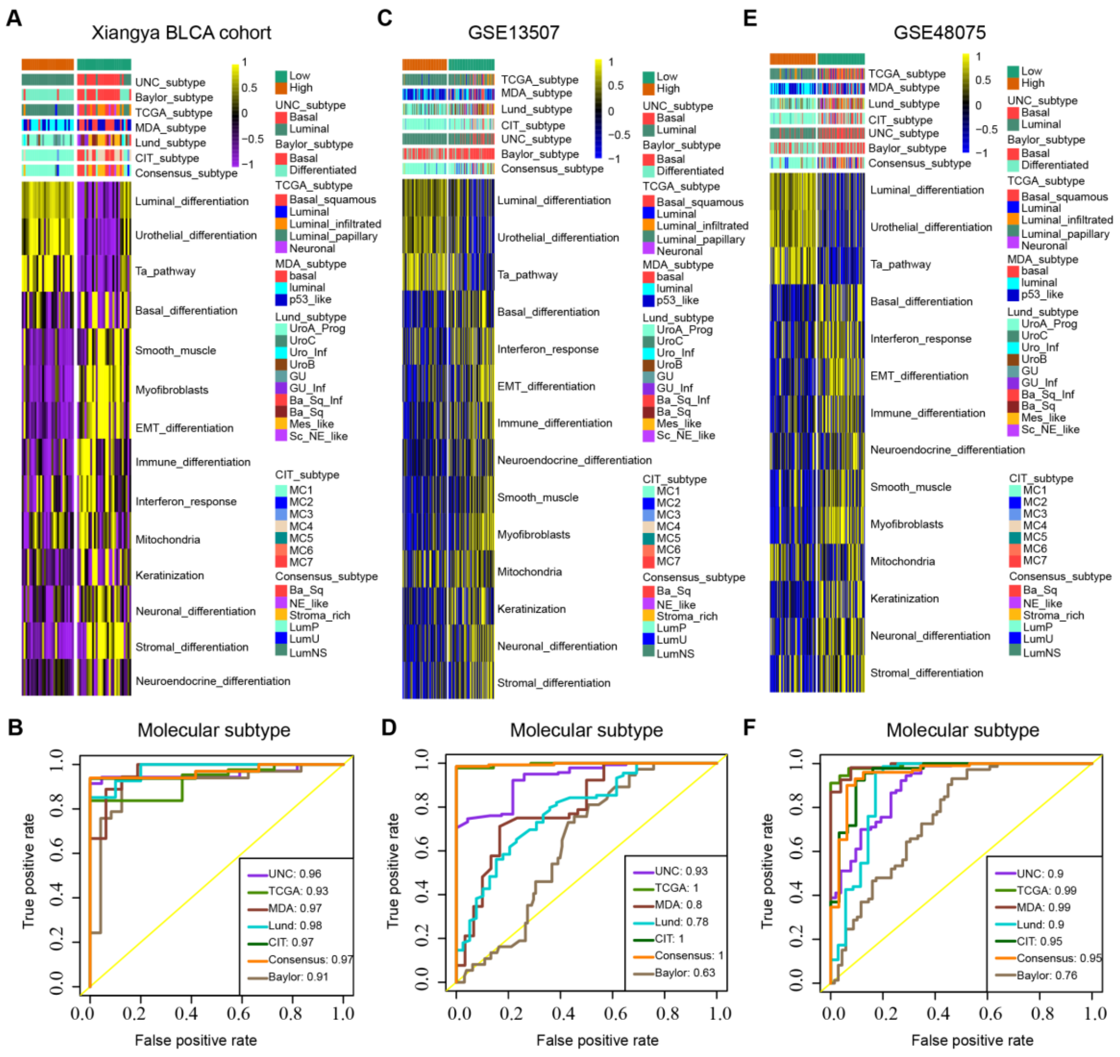

Figure S9. TBX3 predicts molecular typing and guides treatment in the other bladder cancer cohorts. (A, C, E) TBX3 high and low expression of 7 molecular typing results in two groups of patients, and the relationship with the activity of 12 gene sets. (B, D, F) ROC curve evaluated TBX3's accuracy in predicting 7 molecular typing.

Table S1. List of antibodies and primer sequences

1.1Antibodies

Name	Supplier	Cat no.
anti-TBX3	Abcam	ab128854
anti-GAPDH	Abcam	ab8245
anti-TGFβ1	Proteintech	21898-1-AP
anti-FGF2	Abcam	Ab208687
anti-PDGFRA	Abcam	Ab203491
anti-PDGFRB	Abcam	Ab313777
anti-FAP	Abcam	Ab314456
anti-COL4A1	UpingBio	YP-Ab-16983
anti-a-SMA	Proteintech	14395-1-AP
anti-CK19	Abcam	ab52625
anti-CD8	Abcam	ab237709
anti-CD45	BD	557659
anti-CD16/CD32	Biolegend	156603
anti-CD3e	BD	552774
anti-CD4	BD	550954
anti-CD8a	BD	563234
anti-CD31	BD	562614
anti-PDGFRA	eBioscience	17-1401-81
anti-GZMB	BD	563389
Zombie Aqua Fixable Viability Kit	BD	423101

1.2 Primer sequences used in the study

Primers used for ChIP in the TGFβ1 promoter	Primer sequences
binding site sense 1:	5’-GCTCCATTTCCAGGTGTG-3’
binding site antisense1:	5’-CTGGGCACATGGCAAAA-3’
binding site sense 2:	5’-CCTGCTGCTCCGCAACTT-3’
binding site antisense2:	5’-ACCACTGTGCCATCCTCCC-3’
binding site sense 3:	5’-CTCCTGACCCTTCCATCC-3’
binding site antisense3:	5’-GCTGGGAAACAAGGTAGGA-3’
